# Supplementary material for: Vaccines to prevent COVID-19: A living systematic review with Trial Sequential Analysis and network meta-analysis of randomized clinical trials
Source: PLoS One. 2022 Jan 21;17(1):e0260733. doi: 10.1371/journal.pone.0260733 (PMC8782520; doi:10.1371/journal.pone.0260733)
Supplement: S1 File — (DOCX) [file pone.0260733.s001.docx]

**Supplementary**

**to Plos One article entitled**

Vaccines to prevent COVID-19:

a living systematic review with

Trial Sequential Analysis and network meta-analysis of randomized clinical trials

By

Steven Kwasi Korang, MD^1^; Elena von Rohden, MD^1^; Areti Angeliki Veroniki, PhD^2,3^; Giok Ong, PhD^4^; Owen Ngalamika, PhD; Faiza Siddiqui, PhD^1^; Sophie Juul, PhD^1^; Emil Eik Nielsen, MD^1^; Joshua Buron Feinberg, MD^1^;Johanne Juul Petersen, MS^1^; Christian Legart, MS^1,6^; Afoke Kokogho, MD^7^; Mathias Maagaard, MD^1,8^; Sarah Klingenberg, Cand Scient ^1,9^; Lehana Thabane, PhD^10^; Ariel Bardach, PhD^11^; Agustín Ciapponi, PhD^11^; Allan Randrup Thomsen, MD, Dr Med Sci^12^; Janus C Jakobsen, MD, PhD^1,9,13^; Christian Gluud, MD, Dr Med Sci^1,9,13^

Contents

[**Extended method section** 3](#_Toc85368916)

[**Search Strategies** 8](#_Toc85368917)

[**Extended results section** 12](#_Toc85368918)

[**Supplementary figures and tables** 15](#_Toc85368919)

[**RoB2 assessment** 15](#_Toc85368920)

[**Table of excluded studies** 17](#_Toc85368921)

[**Additional supplementary figures** 19](#_Toc85368922)

[**Alternative pooling method (random-effects model versus fixed-effect model)** 46](#_Toc85368923)

[**References** 54](#_Toc85368924)

# **Extended method section**

**Search**

We search the following databases: Cochrane Central Register of Controlled Trials, Medical Literature Analysis and Retrieval System Online (MEDLINE Ovid), Excerpta Medica database (Embase Ovid), Latin American and Caribbean Health Sciences Literature (LILACS; Bireme), Science Citation Index Expanded (SCI-EXPANDED; Web of Science), Conference Proceedings Citation Index—Science (CPCI-S; Web of Science), Chinese Biomedical Literature Database (CBM), China Network Knowledge Information (CNKI), Chinese Science Journal Database (VIP), and Wanfang Database to identify relevant trials. We searched all databases from their inception to the present (May 12, 2021). We also searched special COVID-19 trial sites and preprint servers for health sciences. We also searched online trial registries such as ClinicalTrials.gov ([clinicaltrials.gov](http://clinicaltrials.gov)), the Chinese Clinical Trial Registry ([www.chictr.org.cn](http://www.chictr.org.cn)), the European Medicines Agency (EMA) ([www.ema.europa.eu/](https://www.ema.europa.eu/)), the World Health Organization (WHO) International Clinical Trials Registry Platform ([www.who.int/ictrp/](http://www.who.int/ictrp/)), and the Food and Drug Administration (FDA) ([www.fda.gov/](https://www.fda.gov/)) for ongoing or unpublished trials. We searched for grey literature in the System for Information on Grey Literature in Europe OpenGrey ([www.opengrey.eu](http://www.opengrey.eu)). For detailed search strategies for all electronic searches, see **search strategies**.

No restrictions were imposed regarding publication status, publication year, and language.

We included randomized clinical trials with participants irrespective of prior exposure, age, sex, comorbidities, immune status, and risk group.(1) Any vaccine aiming to prevent COVID-19 and irrespective of dose and duration of administration was eligible. All different vaccines are listed in **Table 1**. Randomized clinical trials with any control group, e.g., vaccine versus placebo, ‘active placebo’, no intervention, another vaccine aiming at preventing COVID-19, or any other ‘active’ comparator, were eligible.(1)

**Data collection**

In this living systematic review, six independent investigators (SKK, ER, GO, CL, JJP, ON) received an updated literature search file and include relevant newly published or unpublished trials once a week. A detailed description of the living systematic review methodology can be found in our protocol.(1)

Eleven authors working in pairs (SKK, ER, GO, FS, JF, EEN, SJ, CL, JJP, AG, AB) independently extracted data from included trials in a predefined form. All the extracted data were double-checked by a third author (SKK, ER, FS, JF, JJP or GO). Disagreements were resolved by discussion or, if required, through consultation with a fourth author (SKK or CG). We contacted the trial authors by email to specify any missing data, which may not be reported sufficiently or not at all in the publication.

**Table S1**: Definition of outcomes

| **Primary outcomes** | **Definition of outcome** |
| --- | --- |
| All-cause mortality | Death of all causes |
| Vaccine efficacy | Either preventing COVID-19 symptoms plus positive polymerase chain rection (PCR) test, preventing severe COVID-19 symptoms plus positive PCR test, or preventing positive PCR test only; and serious adverse events. |
| Serious adverse events | Proportion of participants with one or more serious adverse events. We will use the International Conference on Harmonization of technical requirements for registration of pharmaceuticals for human use—Good Clinical Practice (ICH-GCP) definition of a serious adverse event, which is any untoward medical occurrence that resulted in death, was life-threatening, required hospitalization or prolonging of existing hospitalization, and resulted in persistent or significant disability or jeopardized the participant. If the trialists do not use the ICH-GCP definition, we will include the data if the trialists use the term “serious adverse event.” If the trialists do not use the ICH-GCP definition nor use the term serious adverse event, then we will also include the data if the event clearly fulfills the ICH-GCP definition for a serious adverse event. |
| **Secondary outcomes** |  |
| Health-related quality of life | Health-related quality of life (assessed on any valid continuous scale) |
| Adverse events not considered serious | Proportion of participants with one or more adverse events not considered serious. We will exploratorily assess each type of adverse events not considered serious separately (see below) |

**Trial Sequential Analysis**

When assessing the vaccine efficacy, we planned three trial sequential analyses with a relative risk reduction of 20%, 50%, and 70% respectively. The 50% relative risk reduction was our primary analysis. We used a relative risk reduction of 20% for the remaining dichotomous outcomes, an alpha of 1·67% for all our outcomes, a beta of 10%, and the observed diversity as suggested by the trials in the meta-analysis.(1)

**Network meta-analysis**

We conducted a random-effects network meta-analysis, assuming a common within network heterogeneity for each analysis, since the nature of the interventions in the network are similar. (2,3)
When a random-effects model could not be performed directly due to the small number of studies in a NMA, we used the force option in the network meta command.

Network meta-analysis was performed using Stata 16.1 (command: mvmeta) under the frequentist framework using the network suite of commands.(4,5)

We assessed a priori the two prerequisite assumptions: transitivity and consistency. We assessed for the transitivity assumption across treatment comparisons in the network using boxplots and evaluated the assumption of consistency using the design-by-treatment interaction model as a global test.(6,7) The transitivity assumption for carrying out an network meta-analysis was evaluated using prespecified effect modifiers.(1)

The estimation of each treatment comparison was reported separately using the relevant effect size (risk ratio or efficacy), and the corresponding 95% confidence interval (CI). We used the network forest plot to illustrate the summary effect size of the comparative effectiveness among interventions. Along with the estimated effect sizes, we presented the ranking probabilities for each treatment being at each possible rank, as well as the surface under the cumulative ranking curve (SUCRA).(3,7) A rank-heat plot was used to depict the SUCRA values (and their 95% CI) across all outcomes.(8) A detailed description of our approach to network meta-analysis can be found in our protocol.(1)

**Difference between protocol and review**

We planned to include exploratory outcomes SARS-CoV-2 neutralizing antibody titers, SARS-CoV-2, IgG-binding antibody titers, seroconversion for SARS-CoV-2 neutralizing antibody, seroconversion for SARS-CoV-2 IgG-binding antibody, individual types of serious adverse events, and individual types of non-serious adverse events.(1) These outcomes will be reported in a subsequent systematic review.

As we experienced rare events when assessing all-cause mortality and vaccine efficacy, we used beta-binominal regression for those meta-analyses.(9,10) We pooled participants receiving placebo and MenACWY for the outcome vaccine efficacy as we assumed no effect of this vaccine on the prevention of COVID-19. We did not think this could be justified for the remaining outcomes.

## **Search Strategies**

Cochrane Central Register of Controlled Trials (2021, Issue 5) in the Cochrane Library (1266 hits)

#1 MeSH descriptor: [Coronavirus Infections] explode all trees

#2 ((corona near (virus or viral or infection*)) or coronaviri* or covid* or sars-cov-2)

#3 #1 or #2

#4 MeSH descriptor: [Vaccines] explode all trees

#5 MeSH descriptor: [Vaccination] explode all trees

#6 MeSH descriptor: [Immunization] explode all trees

#7 (vaccin* or immun*)

#8 ((RNA or DNA or recombinant* or vector* or inactivat* or live attenuat* or dendritic or adeno* or encapsulated or plasmid* or protein subunit or peptid* or spike) near/5 vaccin*)

#9 (BNT162 or AZD1222 or Ad5* or INO-4800 or bacTRL* or PRO-nCov-100* or v-sars or vero cell* or SCB-2019 or NVX-COV2373 or BCG vaccin* or VPM1002 or aAPC or AV-COVID-19 or TBD* or Ad26* or MVA* or hAd5* or PiCoVacc or BBIBP-CorV)

#10 #4 or #5 or #6 or #7 or #8 or #9

#11 #3 and #10

**MEDLINE Ovid (1946 to May 2021) (2019 hits)**

1. exp Coronavirus Infections/

2. ((corona adj (virus or viral or infection*)) or coronaviri* or covid* or sars-cov-2).mp. [mp=title, abstract, original title, name of substance word, subject heading word, floating sub-heading word, keyword heading word, organism supplementary concept word, protocol supplementary concept word, rare disease supplementary concept word, unique identifier, synonyms]

3. 1 or 2

4. exp Vaccines/

5. exp Vaccination/

6. exp Immunization/

7. (vaccin* or immun*).mp. [mp=title, abstract, original title, name of substance word, subject heading word, floating sub-heading word, keyword heading word, organism supplementary concept word, protocol supplementary concept word, rare disease supplementary concept word, unique identifier, synonyms]

8. ((RNA or DNA or recombinant* or vector* or inactivat* or live attenuat* or dendritic or adeno* or encapsulated or plasmid* or protein subunit or peptid* or spike) adj5 vaccin*).mp. [mp=title, abstract, original title, name of substance word, subject heading word, floating sub-heading word, keyword heading word, organism supplementary concept word, protocol supplementary concept word, rare disease supplementary concept word, unique identifier, synonyms]

9. (BNT162 or AZD1222 or Ad5* or INO-4800 or bacTRL* or PRO-nCov-100* or v-sars or vero cell* or SCB-2019 or NVX-COV2373 or BCG vaccin* or VPM1002 or aAPC or AV-COVID-19 or TBD* or Ad26* or MVA* or hAd5* or PiCoVacc or BBIBP-CorV).mp. [mp=title, abstract, original title, name of substance word, subject heading word, floating sub-heading word, keyword heading word, organism supplementary concept word, protocol supplementary concept word, rare disease supplementary concept word, unique identifier, synonyms]

10. 4 or 5 or 6 or 7 or 8

11. 3 and 10

12. (randomized controlled trial or controlled clinical trial).pt. or clinical trials as topic.sh. or trial.ti.

13. (random* or blind* or placebo* or meta-analys*).mp. [mp=title, abstract, original title, name of substance word, subject heading word, floating sub-heading word, keyword heading word, organism supplementary concept word, protocol supplementary concept word, rare disease supplementary concept word, unique identifier, synonyms]

14. 11 and (12 or 13)

**Embase Ovid (1974 to May 2021) (2396 hits)**

1. exp coronaviridae/

2. ((corona adj (virus or viral or infection*)) or coronaviri* or covid* or sars-cov-2).mp. [mp=title, abstract, heading word, drug trade name, original title, device manufacturer, drug manufacturer, device trade name, keyword, floating subheading word, candidate term word]

3. 1 or 2

4. exp vaccine/

5. exp vaccination/

6. exp immunization/

7. (vaccin* or immun*).mp. [mp=title, abstract, heading word, drug trade name, original title, device manufacturer, drug manufacturer, device trade name, keyword, floating subheading word, candidate term word]

8. ((RNA or DNA or recombinant* or vector* or inactivat* or live attenuat* or dendritic or adeno* or encapsulated or plasmid* or protein subunit or peptid* or spike) adj5 vaccin*).mp. [mp=title, abstract, heading word, drug trade name, original title, device manufacturer, drug manufacturer, device trade name, keyword, floating subheading word, candidate term word]

9. (BNT162 or AZD1222 or Ad5* or INO-4800 or bacTRL* or PRO-nCov-100* or v-sars or vero cell* or SCB-2019 or NVX-COV2373 or BCG vaccin* or VPM1002 or aAPC or AV-COVID-19 or TBD* or Ad26* or MVA* or hAd5* or PiCoVacc or BBIBP-CorV).mp. [mp=title, abstract, heading word, drug trade name, original title, device manufacturer, drug manufacturer, device trade name, keyword, floating subheading word, candidate term word]

10. 4 or 5 or 6 or 7 or 8 or 9

11. 3 and 10

12. exp randomized controlled trial/

13. exp controlled clinical trial/

14. exp intermethod comparison/

15. exp double blind procedure/

16. (random* or blind* or placebo* or meta-analys*).mp. [mp=title, abstract, heading word, drug trade name, original title, device manufacturer, drug manufacturer, device trade name, keyword, floating subheading word, candidate term word]

17. 12 or 13 or 14 or 15 or 16

18. 11 and 17

**LILACS (Bireme; 1982 to May 2021) (524 hits)**

((corona and (virus or viral or infection$)) or coronaviri$ or covid$ or sars-cov-2) [Words] and (vaccin$ or immun$) or ((RNA or DNA or recombinant$ or vector$ or inactivat$ or live attenuat$ or dendritic or adeno$ or encapsulated or plasmid$ or protein subunit or peptid$ or spike) and vaccin$) or (BNT162 or AZD1222 or Ad5$ or INO-4800 or bacTRL$ or PRO-nCov-100$ or v-sars or vero cell$ or SCB-2019 or NVX-COV2373 or BCG vaccin$ or VPM1002 or aAPC or AV-COVID-19 or TBD$ or Ad26$ or MVA$ or hAd5$ or PiCoVacc or BBIBP-CorV) [Words]

**CINAHL (Ebsco host; May 2021) (1392 hits)**

S12 S10 AND S11

S11 TX (random* or blind* or placebo* or meta-analys*)

S10 S3 AND S9

S9 S4 OR S5 OR S6 OR S7 OR S8

S8 TX (BNT162 or AZD1222 or Ad5* or INO-4800 or bacTRL* or PRO-nCov-100* or v-sars or vero cell* or SCB-2019 or NVX-COV2373 or BCG vaccin* or VPM1002 or aAPC or AV-COVID-19 or TBD* or Ad26* or MVA* or hAd5* or PiCoVacc or BBIBP-CorV)

S7 TX ((RNA or DNA or recombinant* or vector* or inactivat* or live attenuat* or dendritic or adeno* or encapsulated or plasmid* or protein subunit or peptid* or spike) N5 vaccin*)

S6 TX (vaccin* or immun*)

S5 MH immunization

S4 MH vaccines

S3 S1 OR S2

S2 TX ((corona and (virus or viral or infection*)) or coronaviri* or covid* or sars-cov-2)

S1 MH coronavirus

**BIOSIS (Web of Science; 1969 to May 2021) (826 hits)**

#8 #7 AND #6

#7 TI=(random* or blind* or placebo* or meta-analys* or trial*) OR TS=(random* or blind* or placebo* or meta-analys*)

#6 #5 AND #1

#5 #4 OR #3 OR #2

#4 TS=(BNT162 or AZD1222 or Ad5* or INO-4800 or bacTRL* or PRO-nCov-100* or v-sars or vero cell* or SCB-2019 or NVX-COV2373 or BCG vaccin* or VPM1002 or aAPC or AV-COVID-19 or TBD* or Ad26* or MVA* or hAd5* or PiCoVacc or BBIBP-CorV)

#3 TS=((RNA or DNA or recombinant* or vector* or inactivat* or live attenuat* or dendritic or adeno* or encapsulated or plasmid* or protein subunit or peptid* or spike) and vaccin*)

#2 TS=(vaccin* or immun*)

#1 TS=((corona near (virus or viral or infection*)) or coronaviri* or covid* or sars-cov-2)

**Science Citation Index Expanded (1900 to May 2021) and** **Conference Proceedings Citation Index – Science (1990 to May 2021) (Web of Science) (973 hits)**

#8 #7 AND #6

#7 TI=(random* or blind* or placebo* or meta-analys* or trial*) OR TS=(random* or blind* or placebo* or meta-analys*)

#6 #5 AND #1

#5 #4 OR #3 OR #2

#4 TS=(BNT162 or AZD1222 or Ad5* or INO-4800 or bacTRL* or PRO-nCov-100* or v-sars or vero cell* or SCB-2019 or NVX-COV2373 or BCG vaccin* or VPM1002 or aAPC or AV-COVID-19 or TBD* or Ad26* or MVA* or hAd5* or PiCoVacc or BBIBP-CorV)

#3 TS=((RNA or DNA or recombinant* or vector* or inactivat* or live attenuat* or dendritic or adeno* or encapsulated or plasmid* or protein subunit or peptid* or spike) and vaccin*)

#2 TS=(vaccin* or immun*)

#1 TS=((corona near (virus or viral or infection*)) or coronaviri* or covid* or sars-cov-2)

# **Extended results section**

**All-cause mortality**

**mRNA vaccines**

Two trials reported all-cause mortality. Beta-binominal regression suggested no evidence of a difference (OR, 0·57, 95% CI 0·17 to 1·95; p=0·372; 73 603 participants). The results were similar to our pairwise comparisons reported in the main manuscript.

Risk of bias for this outcome was at overall some concern.

**Viral vector vaccines**

Two trials reported all-cause mortality. Beta-binominal regression suggested that viral vector vaccines reduce mortality (OR, 0·28, 95% CI 0·11 to 0·73; p=0.009; 65 645 participants). The results were similar to our pairwise comparisons reported in the main manuscript.

The subgroup analysis assessing vested interests and the effects of the different viral vector vaccines showed no evidence of differences (p=0·20 and p=0·44) (**Supplement**).

**Vaccine efficacy**

**mRNA vaccines**

Two trials reported vaccine efficacy on symptomatic COVID-19. Beta-binominal regression suggested evidence of a beneficial effects of the mRNA vaccines (OR, 0·05, 95% CI 0·03 to 0·08; p<0·001; 69 285 participants). The results were similar to our pairwise comparisons reported in the main manuscript.

Trial Sequential Analysis showed that we had enough information to confirm that the mRNA vaccines had efficacy of 70% or more (TSA-adjusted CI 89% to 97%) (Appendix). 'Worst‐best' case meta‐analyses showed that incomplete outcome data bias had the potential to influence the results (Appendix).

**Viral vector vaccines**

Four trials reported vaccine efficacy on symptomatic COVID-19. Beta-binominal regression suggested evidence of a beneficial effects of the viral vector vaccines (OR 0·26, 95% CI 0·18 to 0·36; p<0·001; 70 865 participants). The results were similar to our pairwise comparisons reported in the main manuscript.

**Prevention of severe symptoms of COVID-19 plus positive PCR**

**mRNA vaccines**

Two trials reported vaccine efficacy on severe COVID-19. Meta-analysis (RE) showed that mRNA vaccines may have a large effect on preventing severe COVID-19 compared with placebo (efficacy, 93%, 95% CI 99% to -5%; p=0∙05; I^2^ = 55∙7%; 70 719 participants; low certainty of evidence; **Figure S23**). Visual inspection of the forest plot and measures to quantify heterogeneity indicated some signs of heterogeneity.

Trial Sequential Analysis showed that we did not have enough information to confirm or reject that the mRNA vaccines had a vaccine efficacy of 20% or more (**Figure S24)**. Bayes factor (0.03) was below the Bayes factor threshold for significance of 0·1.

**Viral vector vaccines**

Three trials reported vaccine efficacy on severe COVID-19. Meta-analysis (RE) showed that viral vector vaccines may have a large effect on preventing severe COVID-19 compared with placebo (efficacy, 91%, 95% CI 20% to 99%; p=0∙03; I^2^=58·8%; 59 187 participants; low certainty of evidence; **Figure S25**). Visual inspection of the forest plot and measures to quantify heterogeneity indicated substantial heterogeneity.

Trial Sequential Analysis confirmed that we did not have enough information to confirm that the viral vector vaccines had a vaccine efficacy of 20% or more (**Figure S26)**. Bayes factor (0.88) was above the Bayes factor threshold for significance of 0·1.

**Prevention of COVID-19 defined as having a positive PCR test only**

**mRNA Vaccines**

Only one of the trials assessing mRNA vaccines included data on participants with COVID-19 regardless of symptoms, hence we were not able to conduct meta-analysis on this outcome (11).

**Viral vector vaccines**

None of the trials assessing viral vector vaccines reported data on participants with COVID-19 regardless of symptoms, hence we were not able to conduct meta-analysis on this outcome.

# **Supplementary figures and tables**

## **RoB2 assessment**

**-Traffic Light Plot**

**
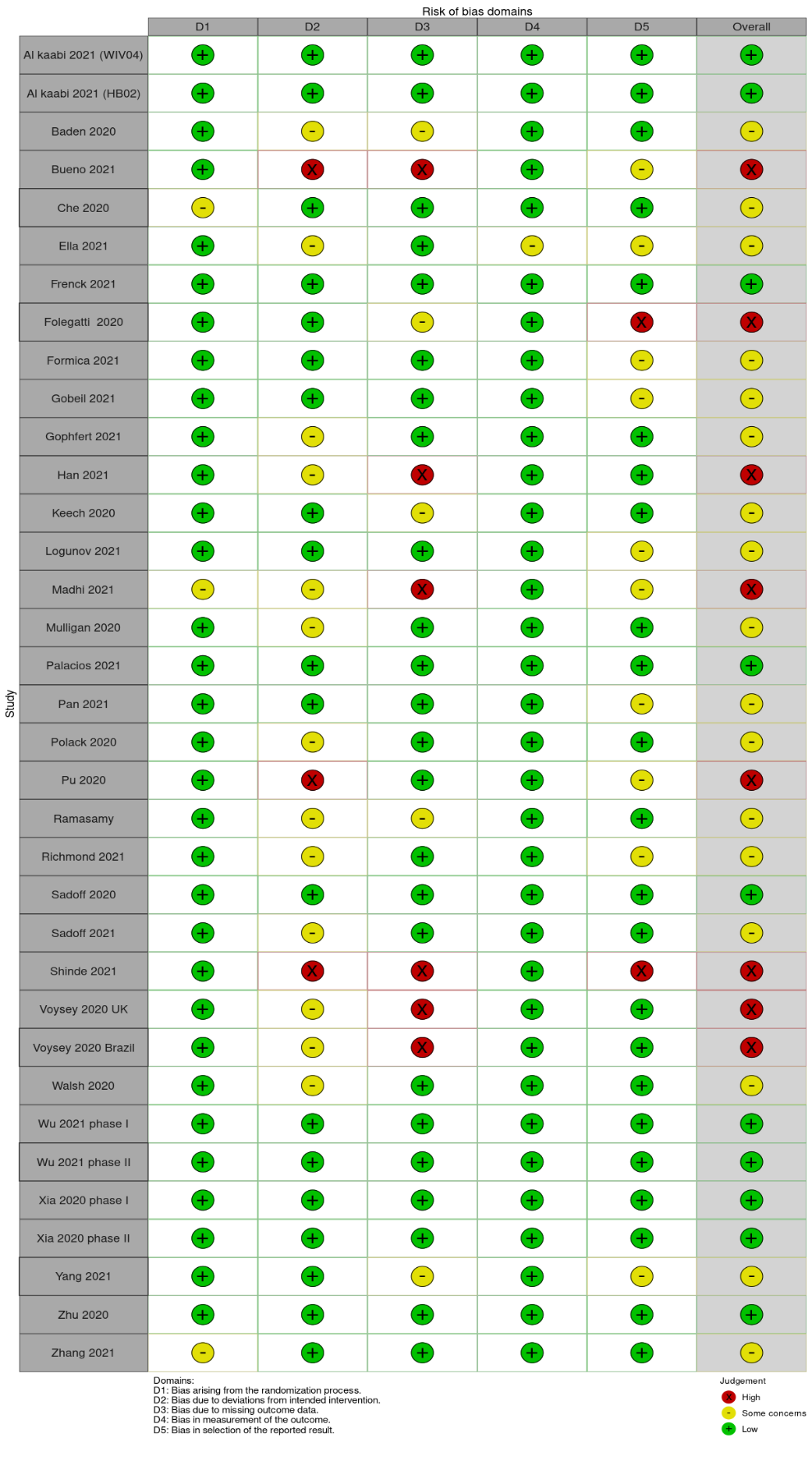
**

**Summery Plot:**

**
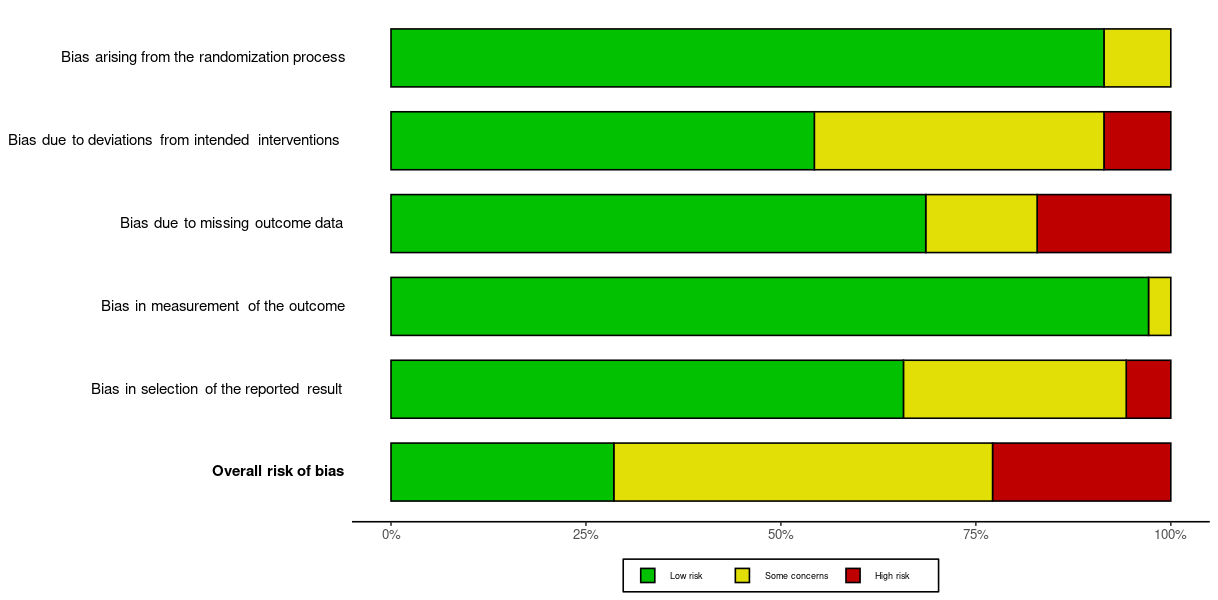
**

## **Table of excluded studies**

| **Study id** | **Reason for exclusion** |
| --- | --- |
| Voysey 2021 | Duplicate |
| Ella 2021 | Duplicate |
| Baden 2020 | Duplicate |
| Voysey 2020 | Duplicate |
| Zhang 2020 | Duplicate |
| Voysey 2021 | Duplicate |
| Xia 2021 | Duplicate |
| Gupta 2019 | Wrong patient population |
| Folegatti 2020 | Duplicate |
| Zhu 2020 | Duplicate |
| Sonani 2021 | Not an RCT |
| Folegatti 2020 | Duplicate |
| NCT04400838 | Duplicate |
| Mahase 2020a | Not an RCT |
| ACTRN12620000674932 | Duplicate |
| NL8547 | Duplicate |
| Giamarellos-Bourboulis 2020 | Wrong indication |
| ISRCTN90906759 | Duplicate |
| Caddy 2020 | Not an RCT |
| Bar-Zeev 2020 | Not an RCT |
| Mulligan 2020 | Duplicate |
| Gupta 2020 | Not an RCT |
| Mahase 2020b | Not an RCT |
| Schachar 2020 | Not an RCT |
| Lawton 2020 | Not an RCT |
| Zhu 2020 | Duplicate |

## **Additional supplementary figures**

**All-cause mortality**

**Figure S1: Subgroup analysis of types of mRNA vaccines on all-cause mortality**


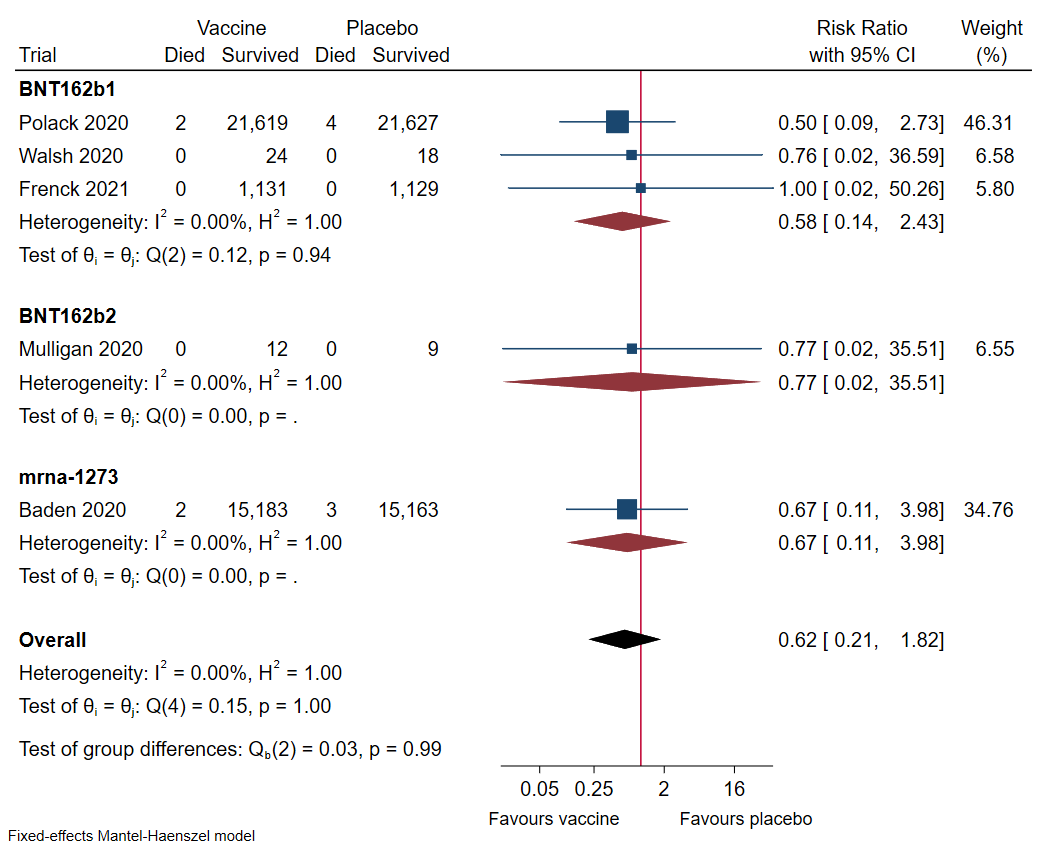


**Figure S2: Trial Sequential Analysis of** **viral vector vaccines versus control on all-cause mortality**


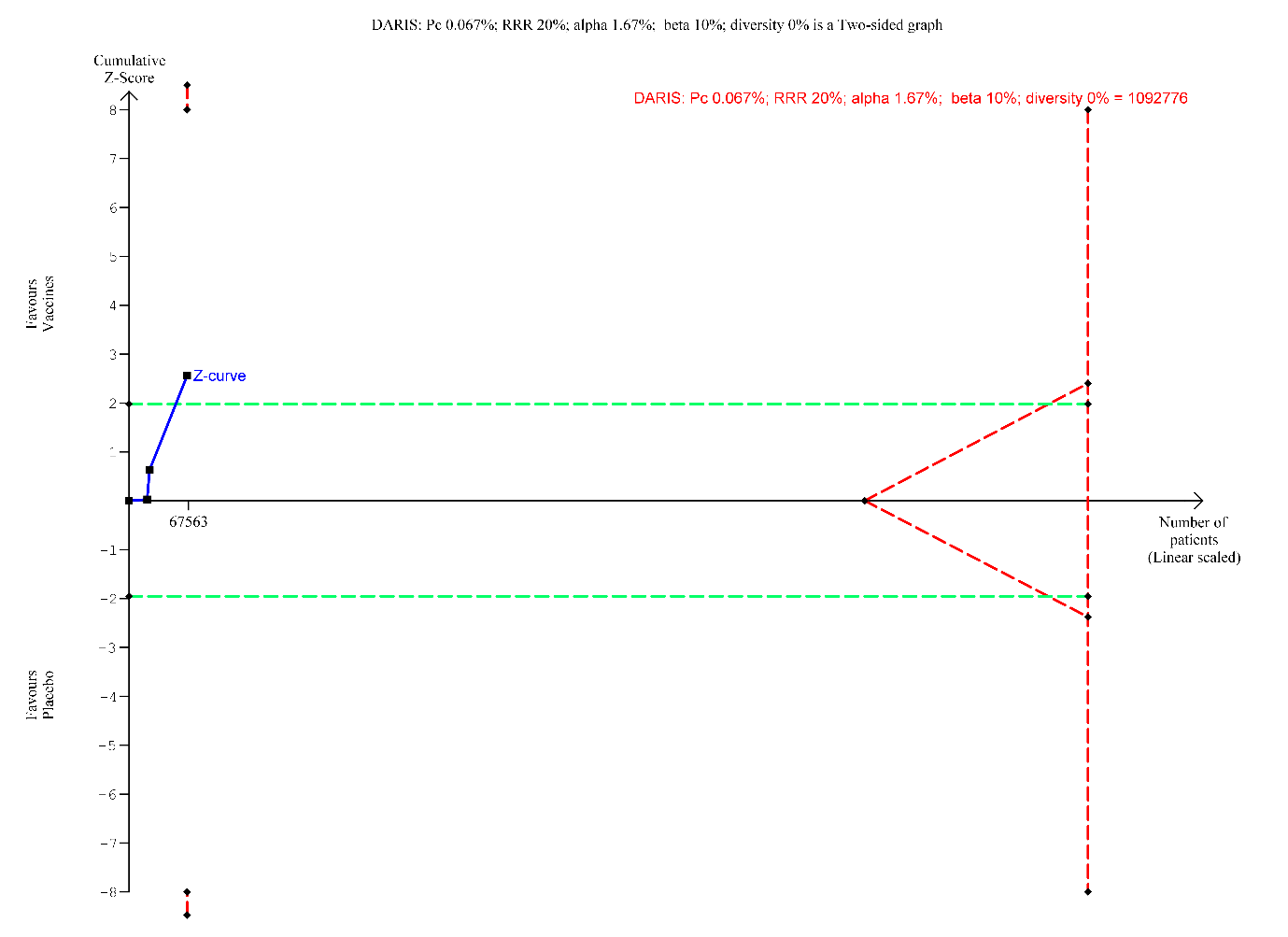


**Figure S3: Subgroup analysis based on vested interests of viral vector vaccines versus control on all-cause mortality**


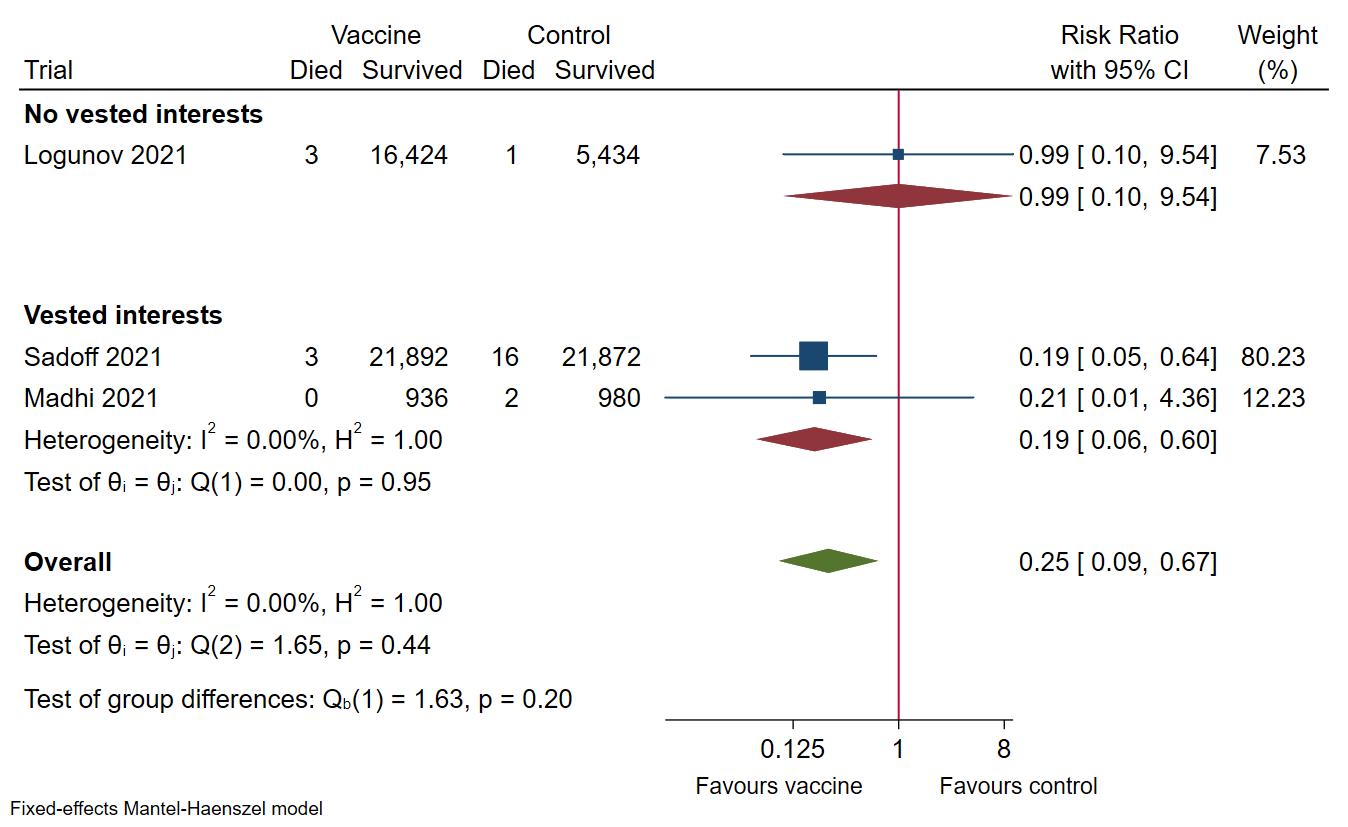


**Figure S4: Subgroup analysis based on type of viral vector vaccine on all-cause mortality**


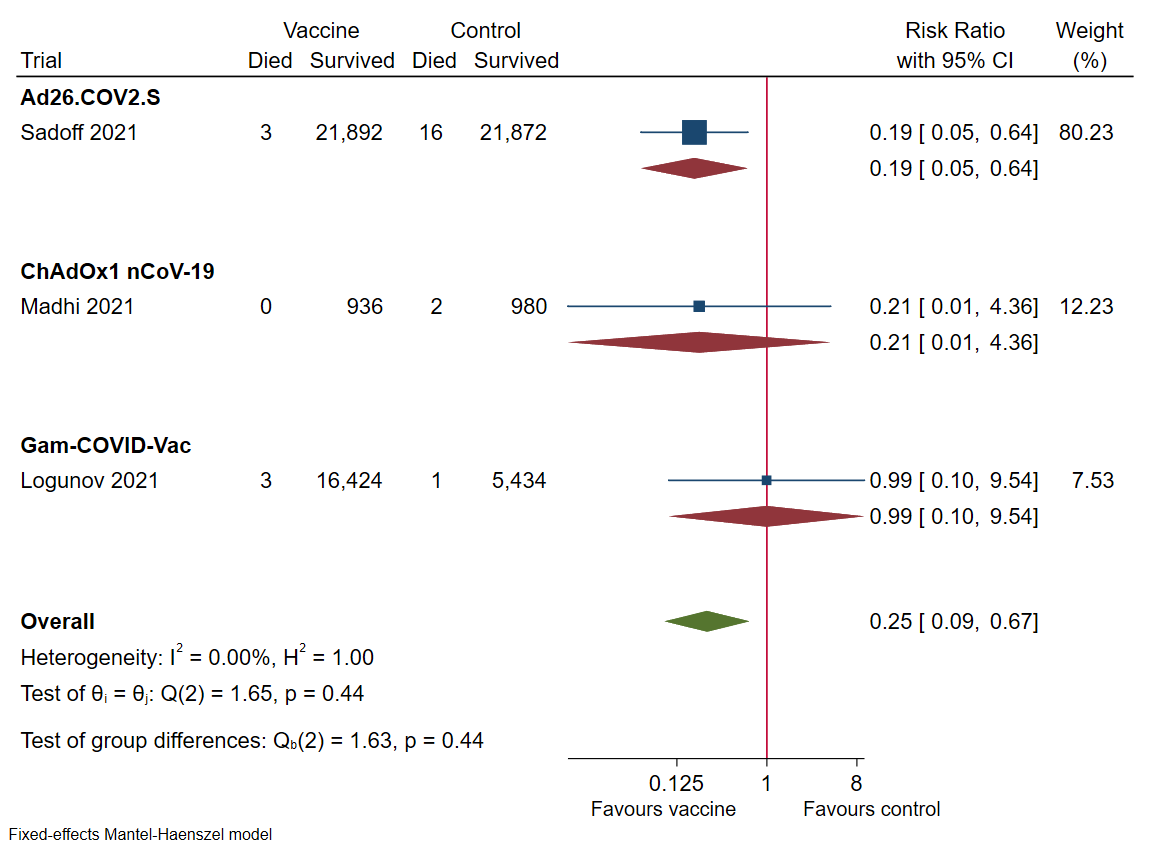


**Figure 5:** **Best-worst sensitivity analysis of mRNA and viral vector vaccines versus control on all-cause mortality**

**mRNA:**


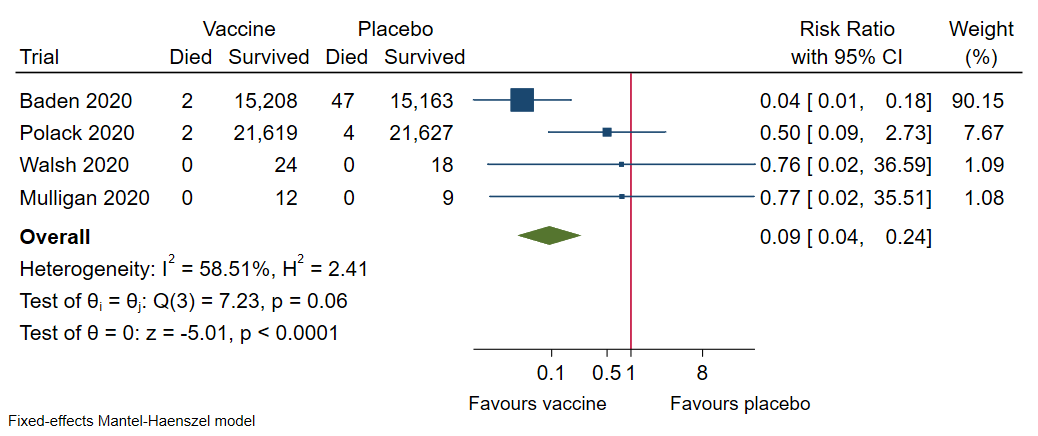


**Viral vector:**


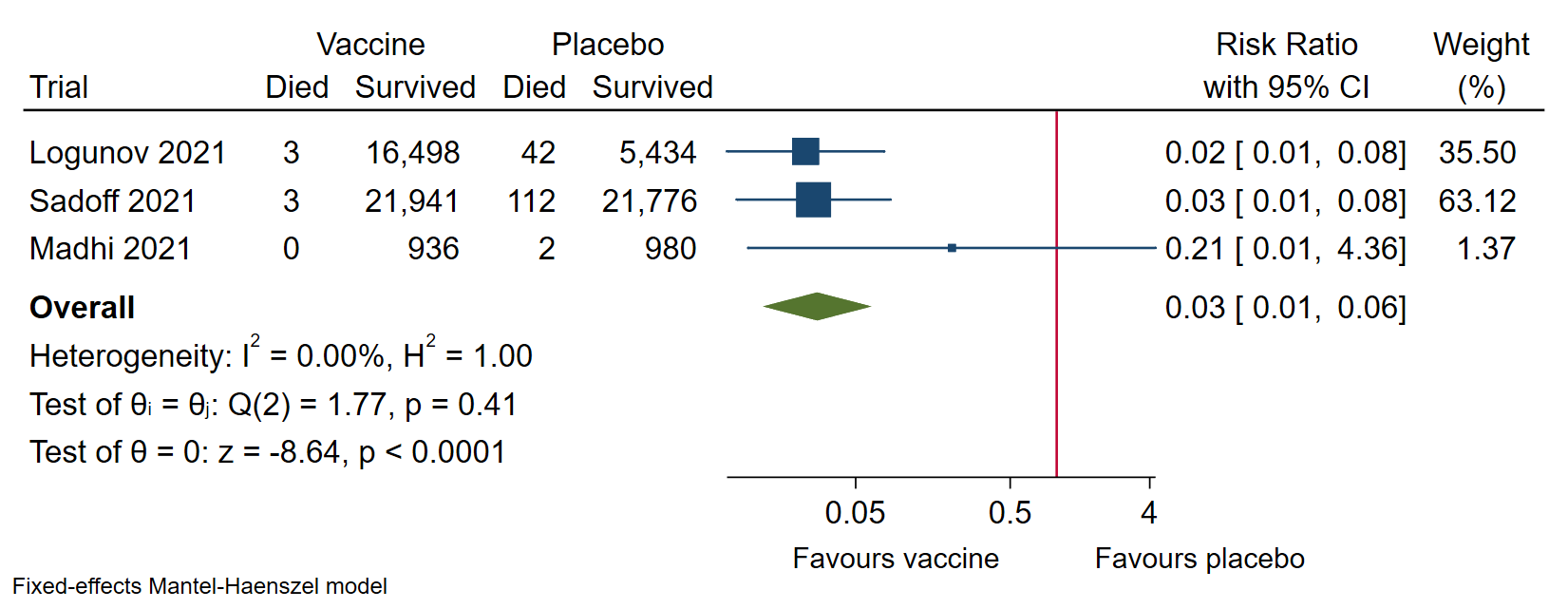


**Figure S6a**: **Network plot for all-cause mortality**


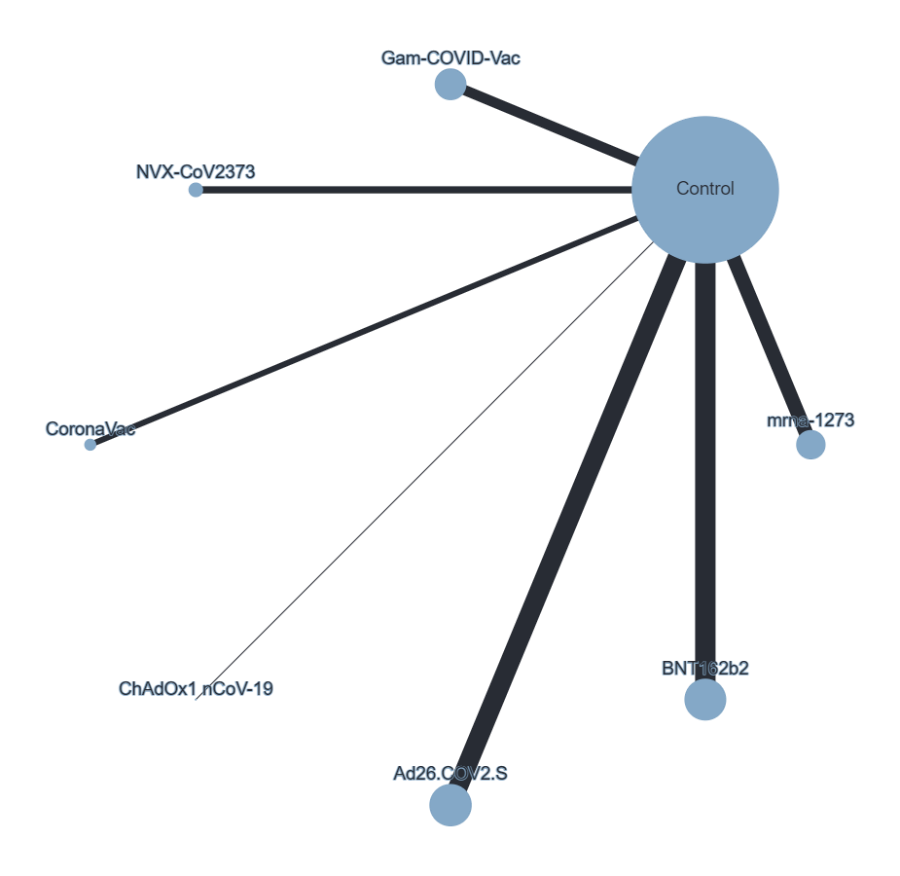


**Figure S6b**: **Network plot for all-cause mortality including visual CINeMA assessment**

Node colour: Risk of bias Edge colour: Indirectness


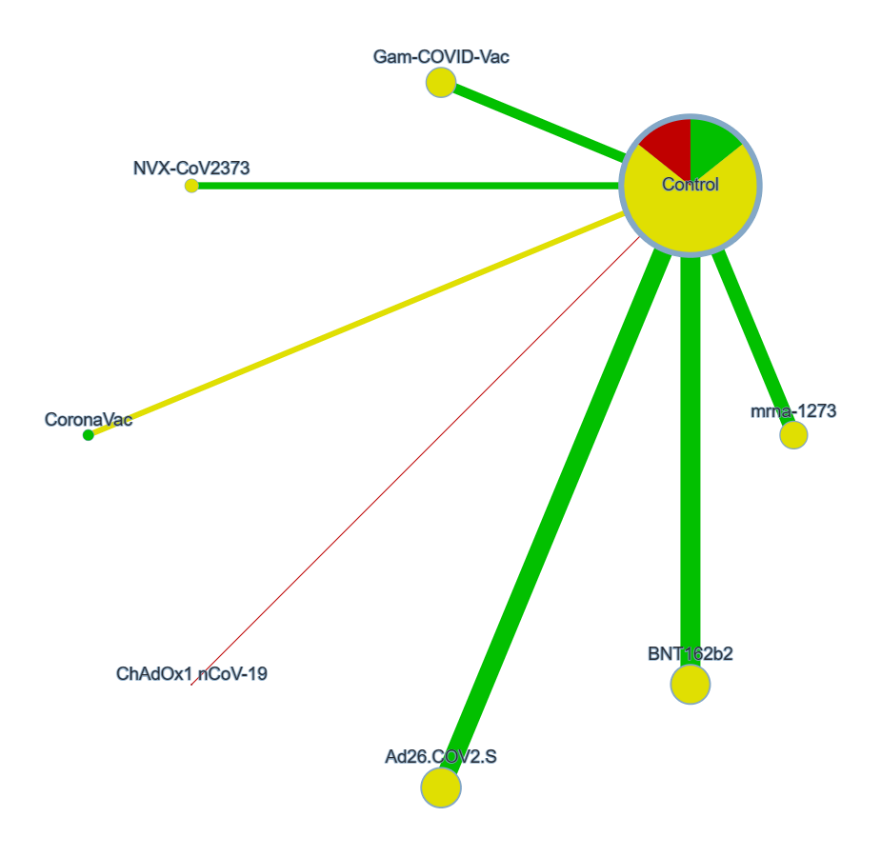


**Figure S6c: CINeMA report on vaccine efficacy for all-cause mortality (see attached)**

**Figure S7**: **Rank of vaccines on mortality**


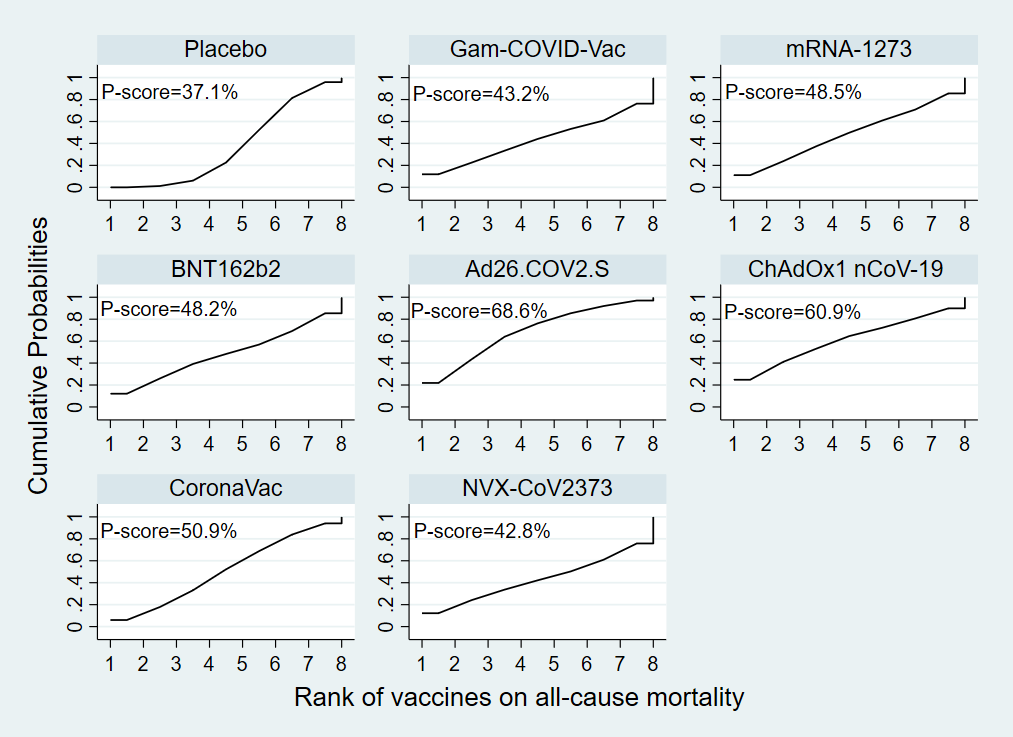


**Figure S8**: **COVID-19 vaccines on all-cause mortality**


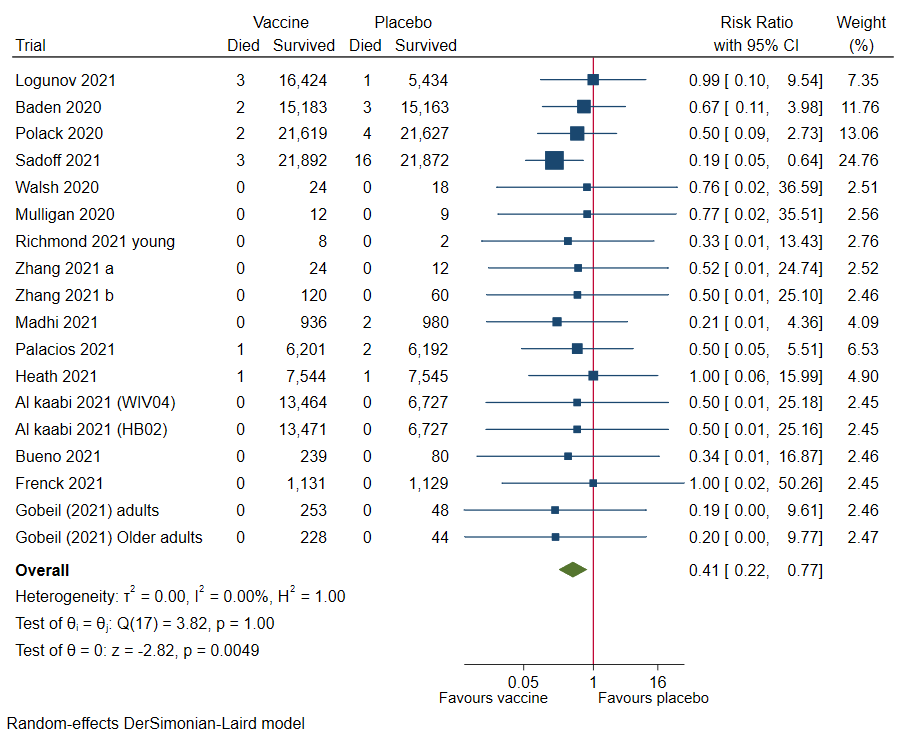


**Figure S9: Trial Sequential Analysis of** **COVID-19 vaccines versus placebo on all-cause mortality**


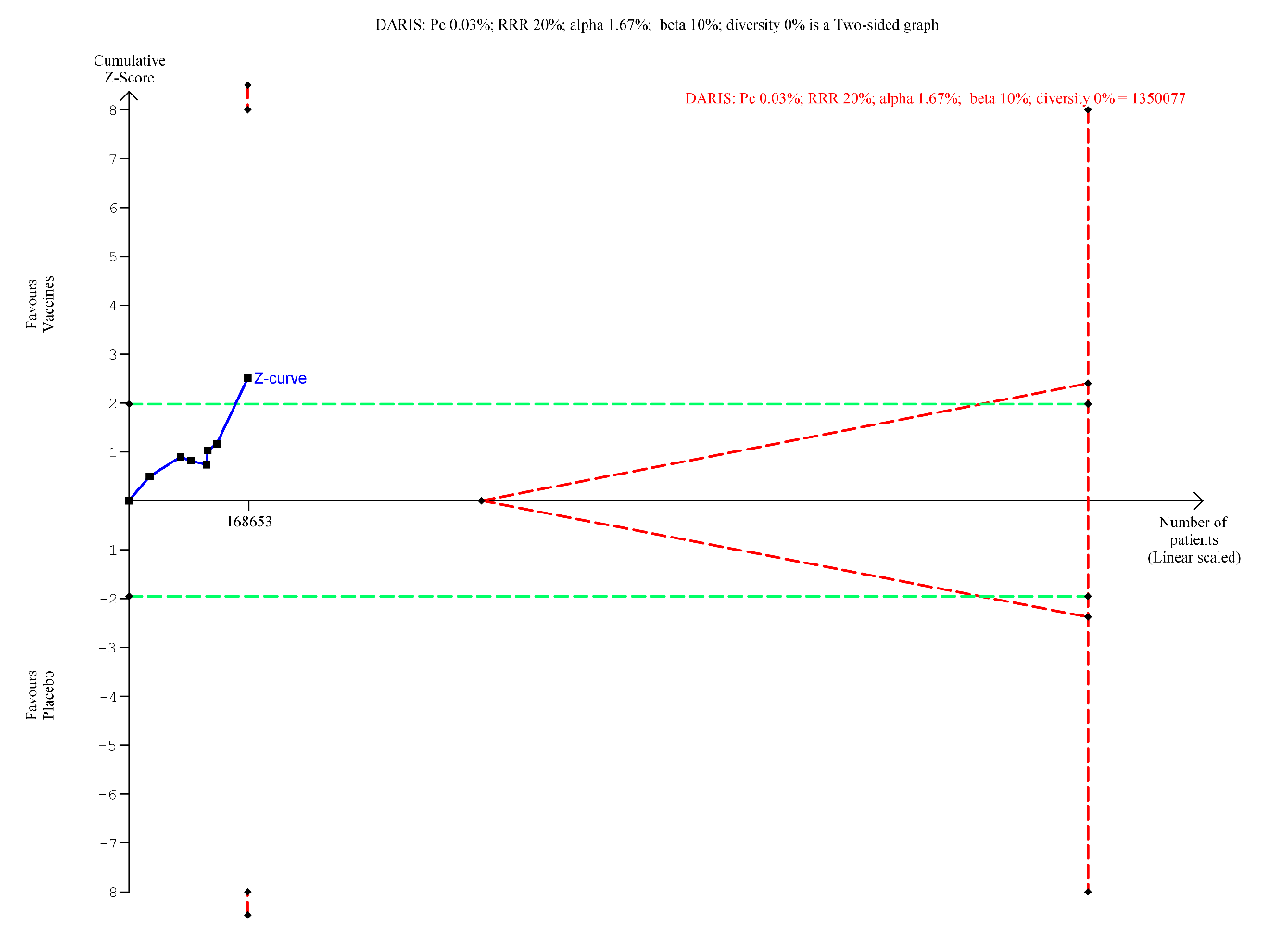


**Vaccine efficacy on symptomatic COVID-19 plus positive PCR**

**Figure S10:** **Trial Sequential Analysis of inactivated vaccine efficacy on symptomatic COVID-19 plus positive PCR**


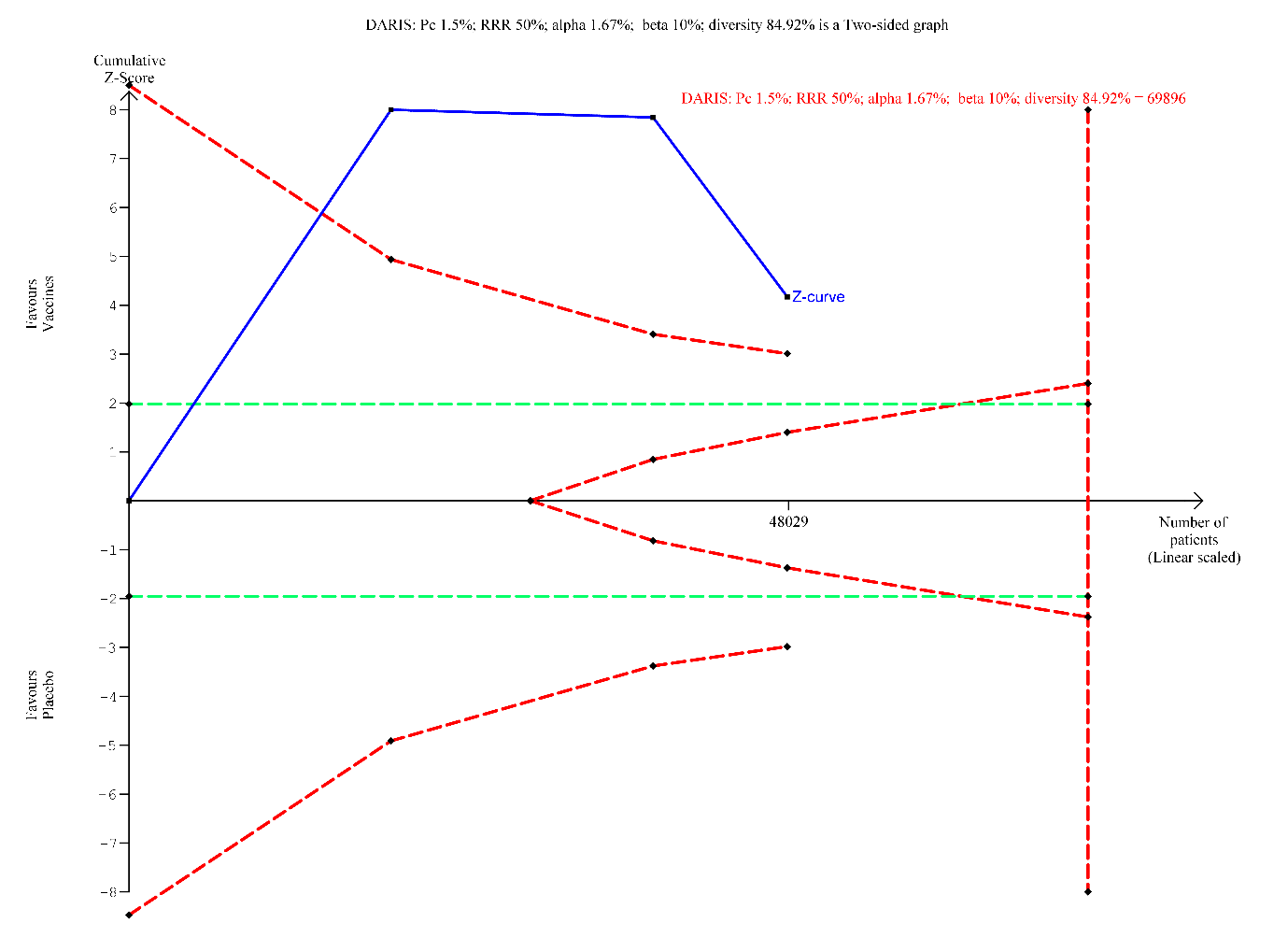


**Figure S11:** **Trial Sequential Analysis of mRNA vaccine efficacy on symptomatic COVID-19 plus positive PCR**


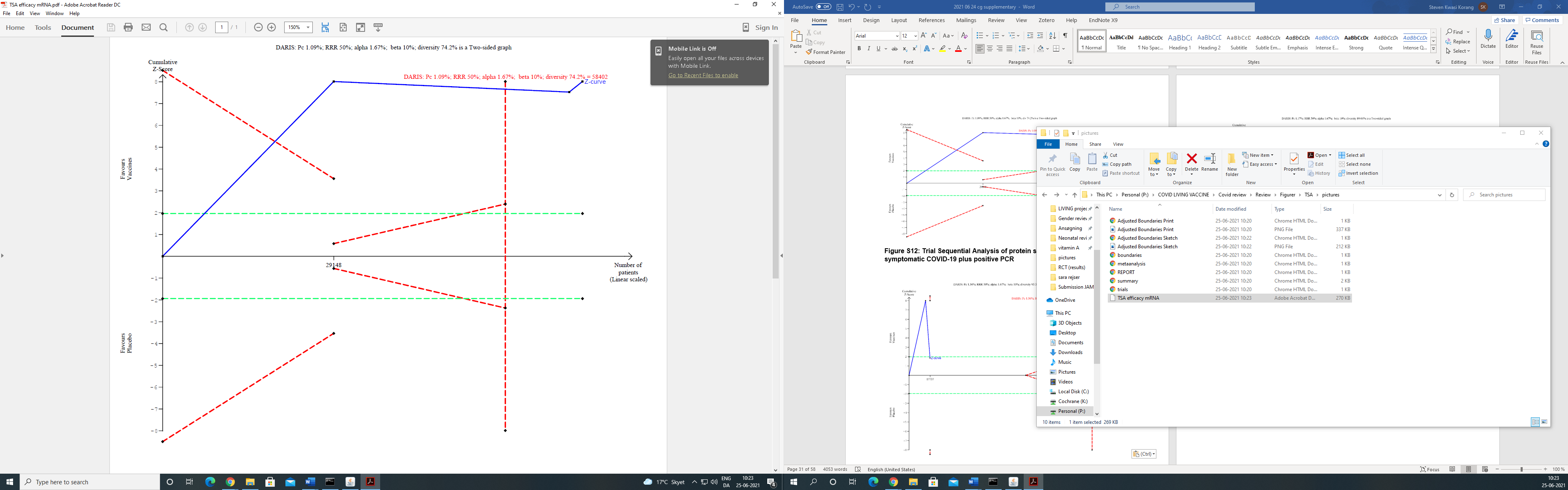


**Figure S12:** **Trial Sequential Analysis of protein subunit vaccine efficacy on symptomatic COVID-19 plus positive PCR**


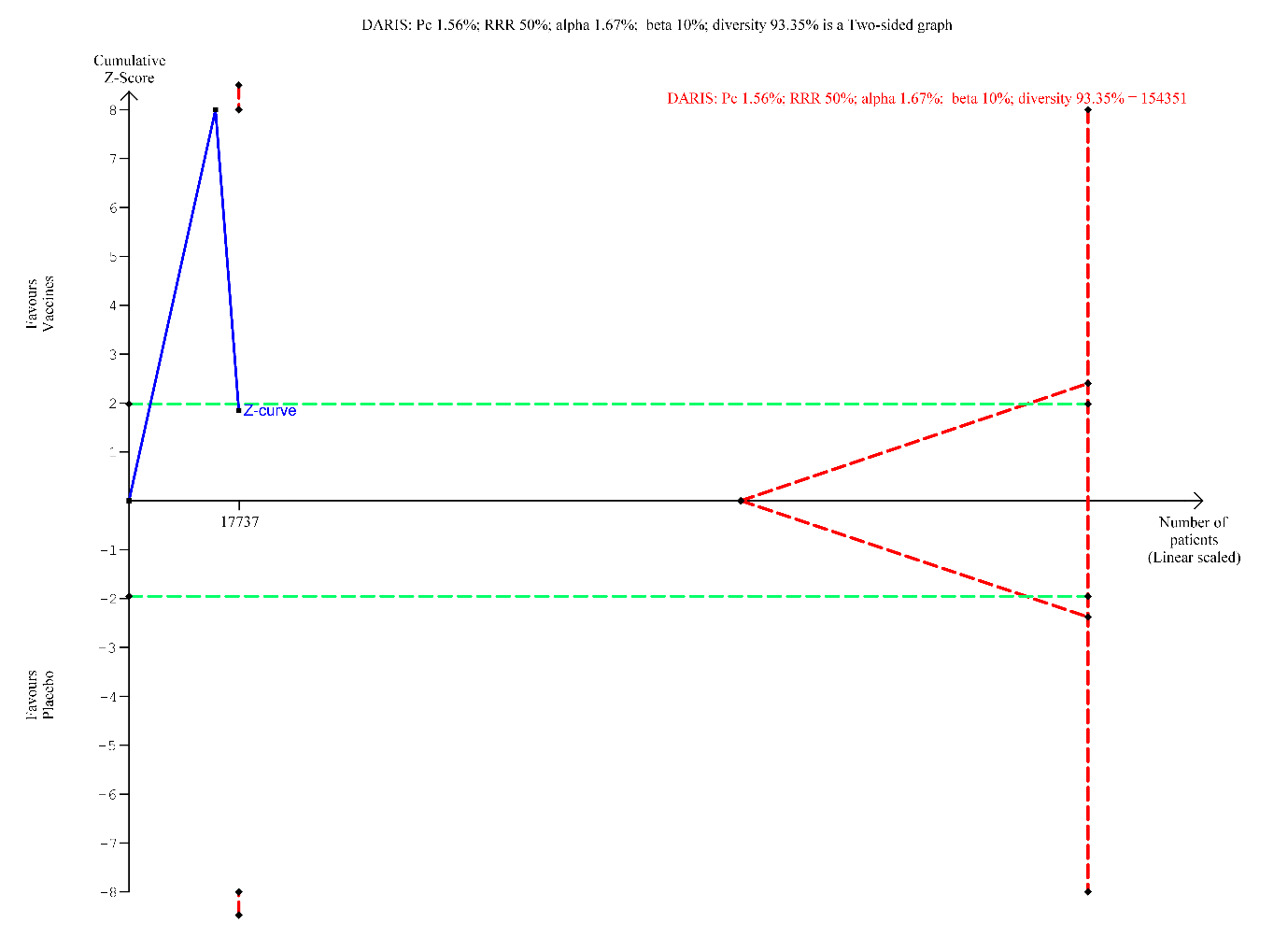


**Figure S13:** **Trial Sequential Analysis of viral vector vaccine efficacy on symptomatic COVID-19 plus positive PCR**


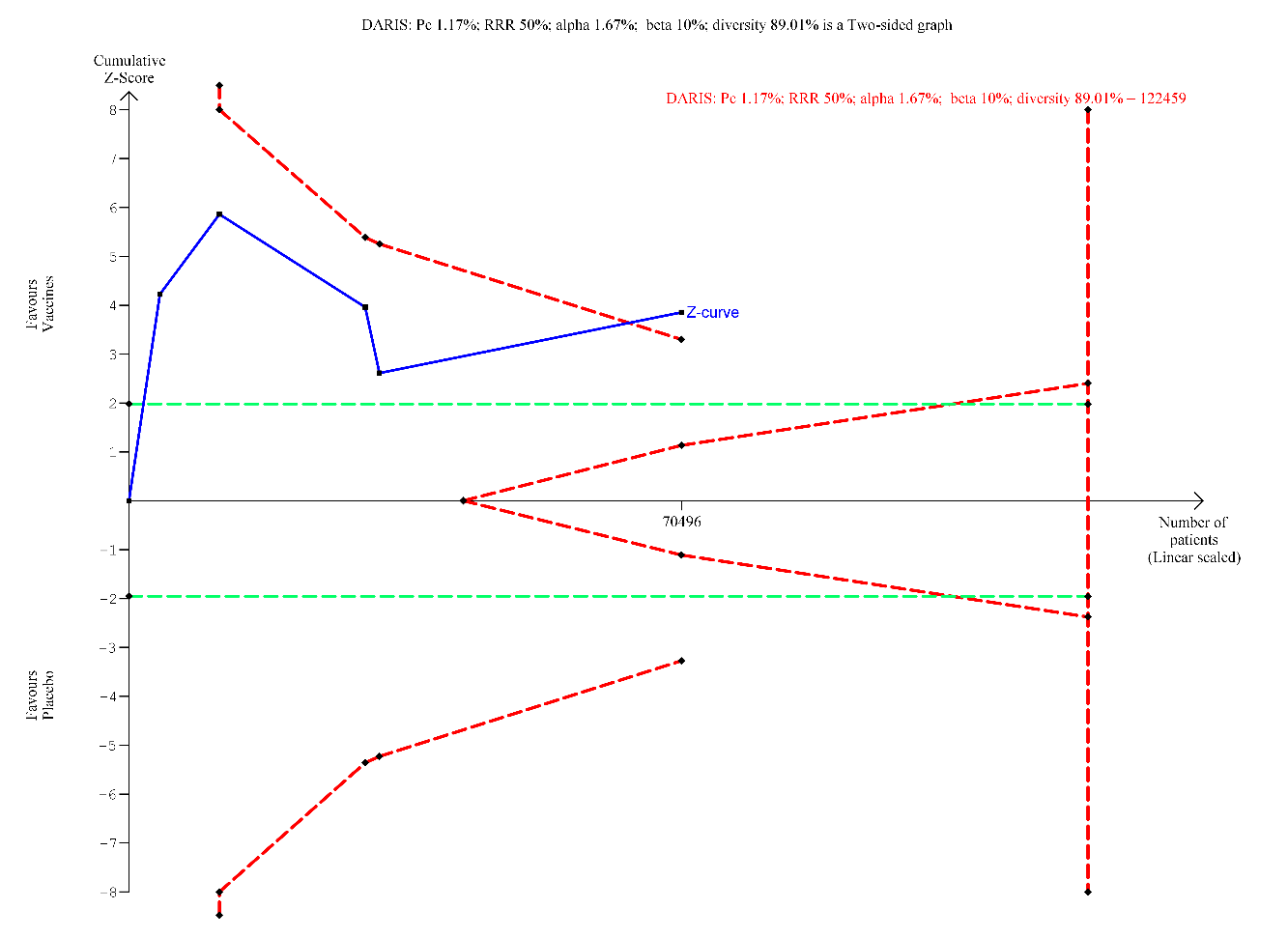


**Figure S14: Subgroup analysis of efficacy of different viral vector vaccines on symptomatic COVID-19 plus positive PCR test**


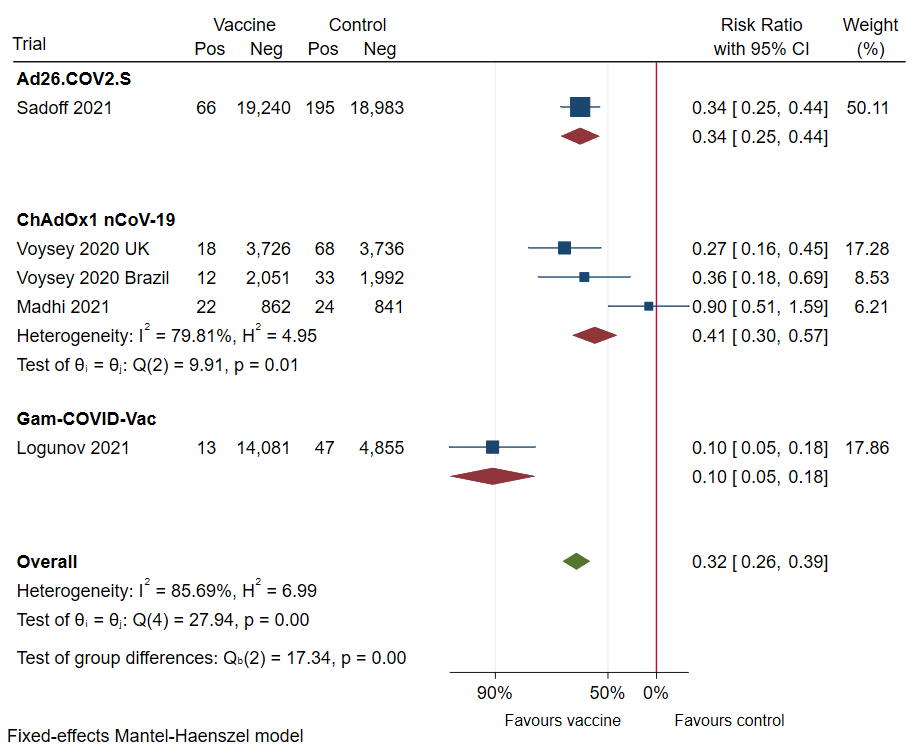


**Figure S15: Subgroup analysis based on vested interests of efficacy of viral vector vaccines on symptomatic COVID-19 plus positive PCR test**


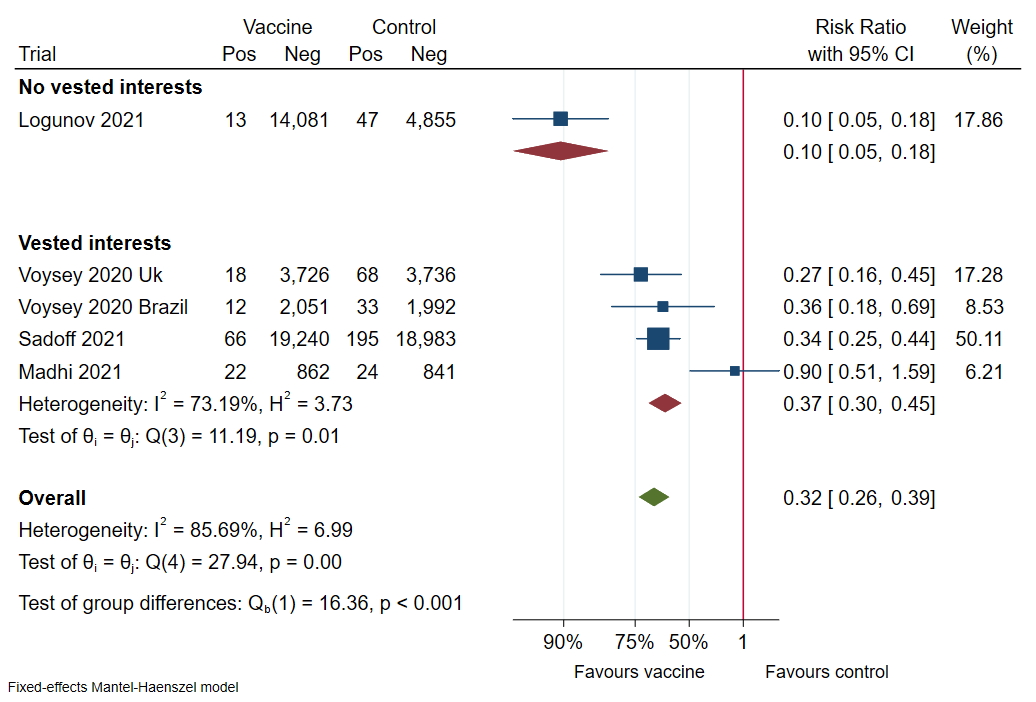


**Figure S16a**: **Network plot of vaccine efficacy on symptomatic COVID-19**


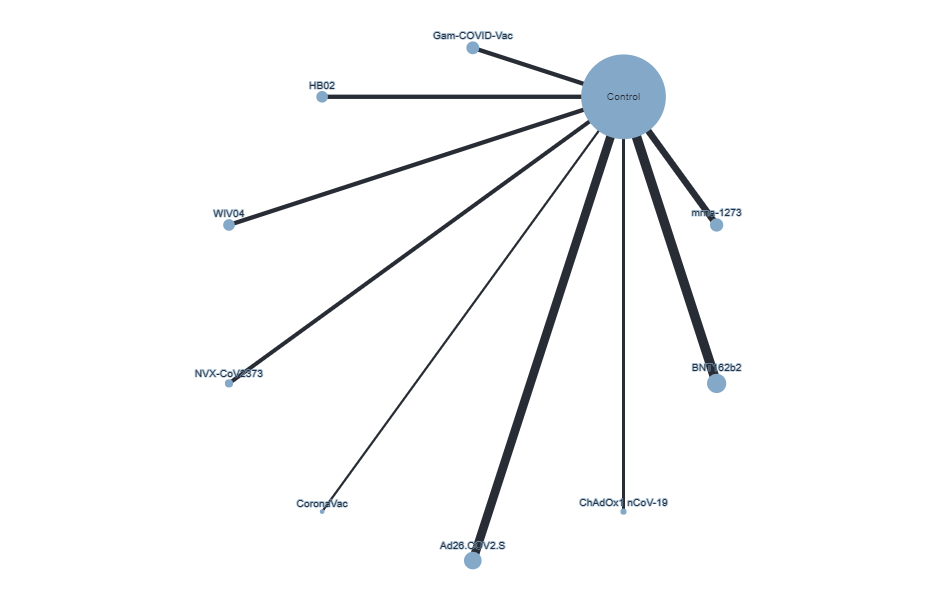


**Figure S16b**: **Network plot of vaccine efficacy on symptomatic COVID-19 including visual CINeMA assessment**

Node colour: Risk of bias Edge colour: Average indirectness


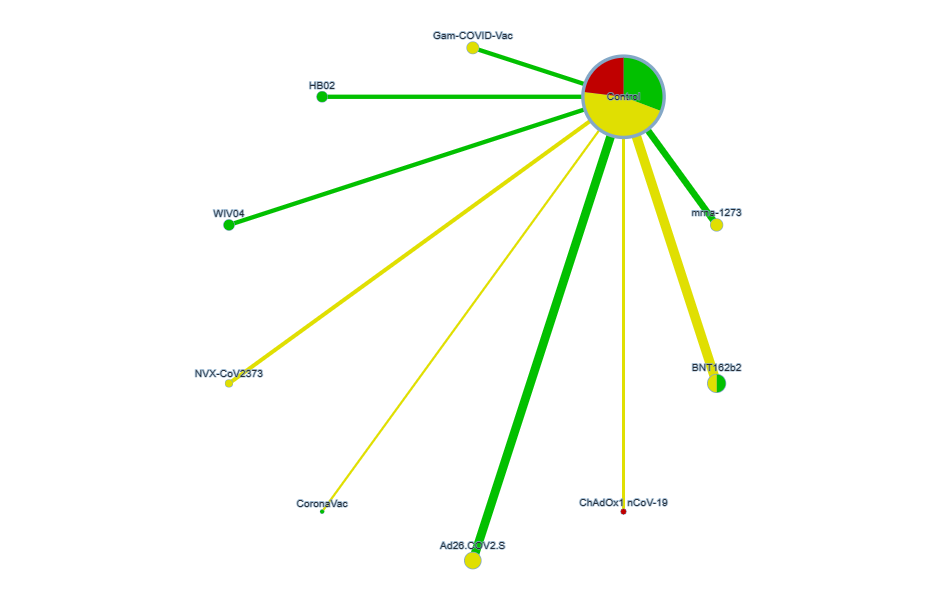


**Figure S16c: CINeMA report on vaccine efficacy for symptomatic COVID-19 (see attached)**

**Figure S16d**: **Rank of vaccines on vaccine efficacy for symptomatic COVID-19**

**Figure S16e**: **Intervalplot on vaccine efficacy for symptomatic COVID-19**

(A=Placebo, B=Gam-COVID-Vac, C=mRNA-1273, D=BNT162b2, E=ChADOx1 nCOV-19, F= Ad26.COV2.S, G=CoronaVac, H=NVX-CoV2373, I=Vero Cell)

**Figure S17: Inactivated vaccines versus placebo on serious adverse events**


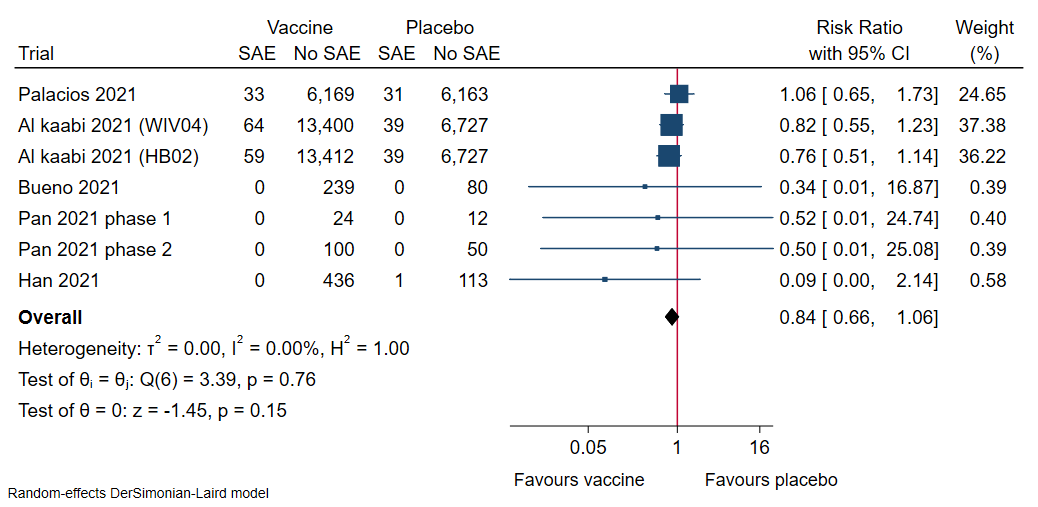


**Figure S18: Trial Sequential Analysis of inactivated vaccines on serious adverse events**


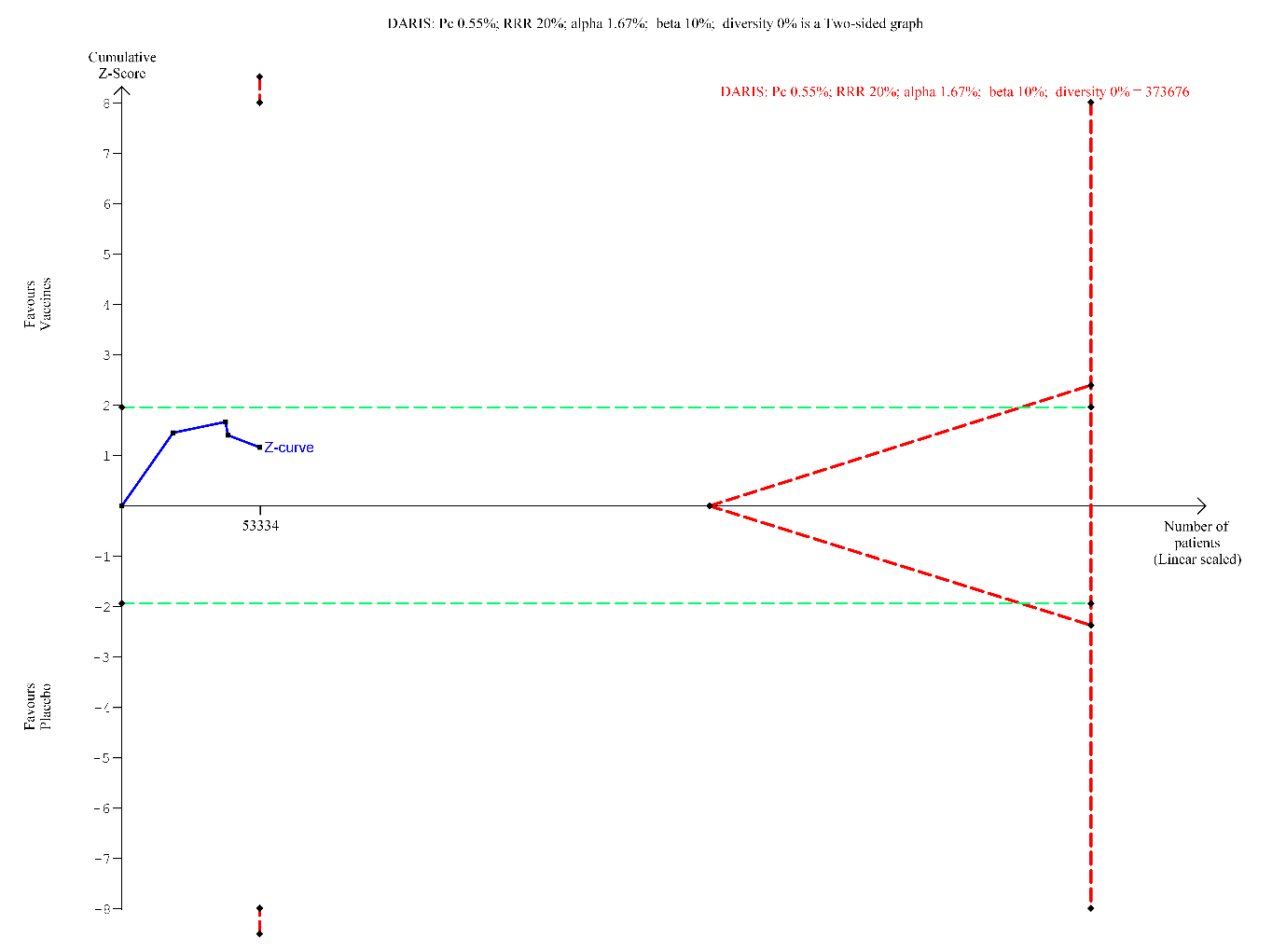


**Figure S19: mRNA vaccines versus placebo on serious adverse events**


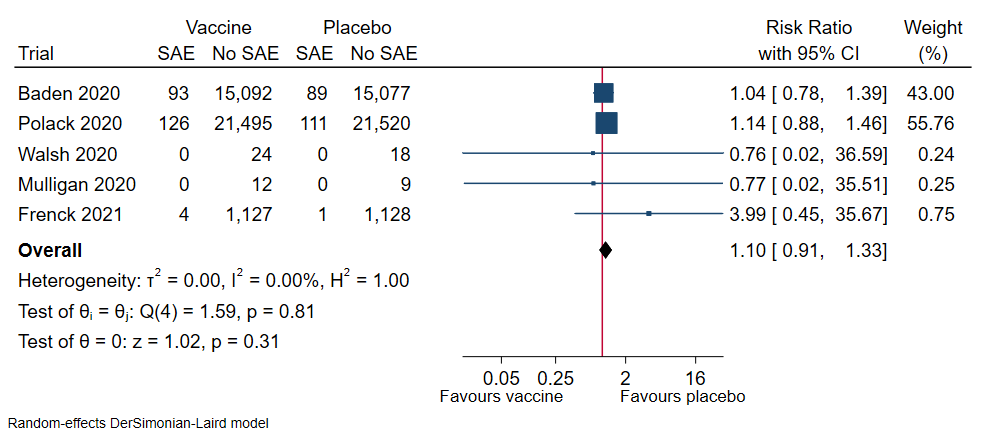


**Figure S20: Trial Sequential Analysis of mRNA vaccines on serious adverse events**


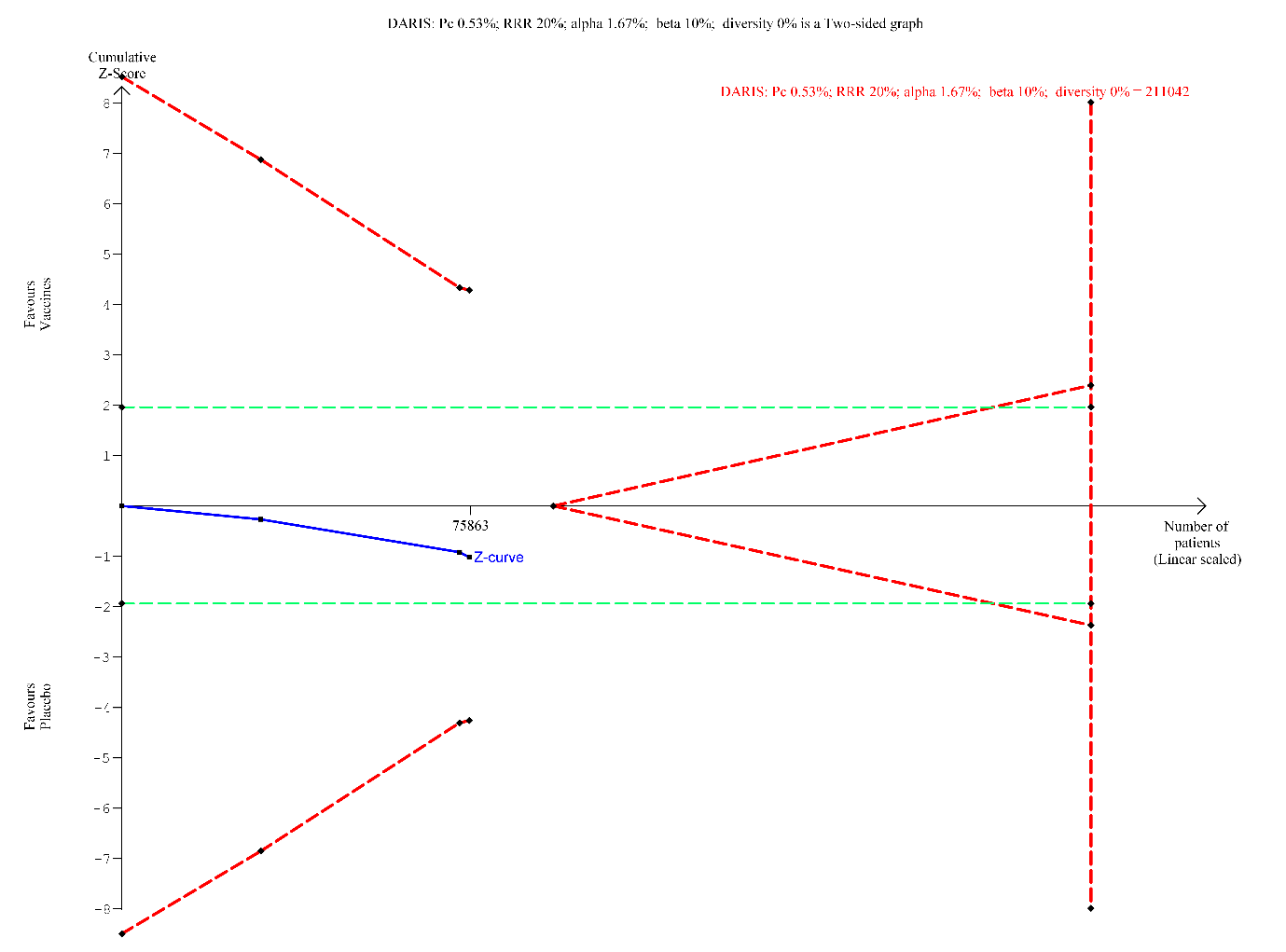


**Figure S21: Protein subunit vaccines versus placebo on serious adverse events**


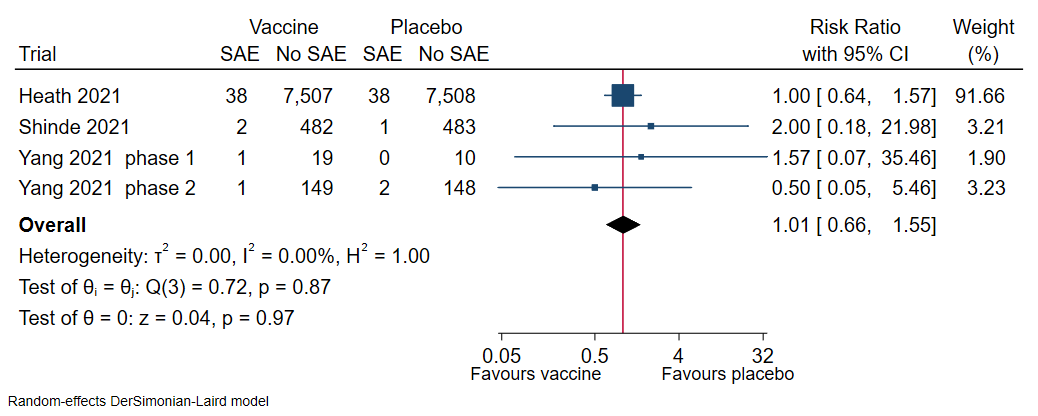


**Figure S22: Trial Sequential Analysis of protein subunit vaccines on serious adverse events**


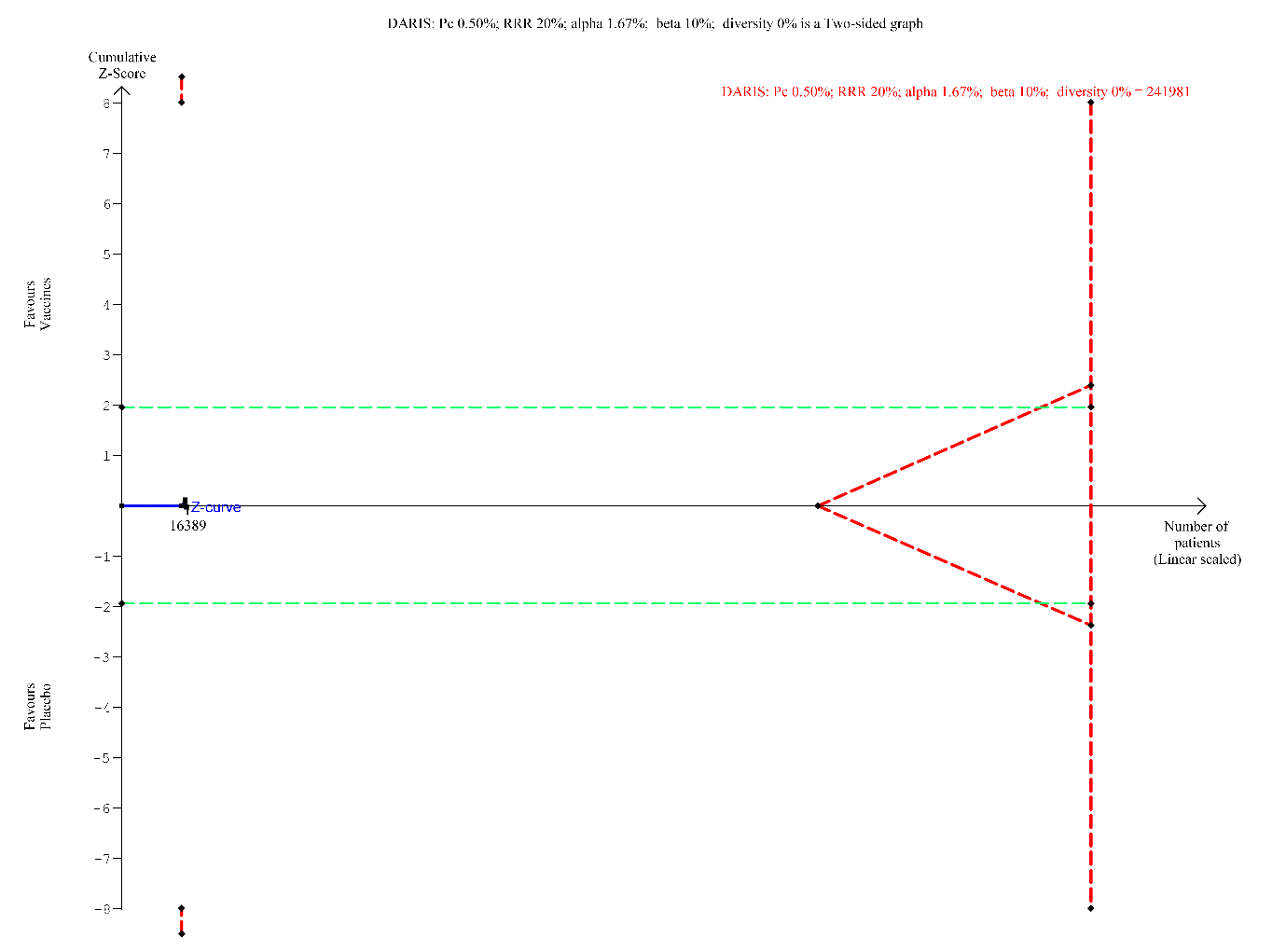


**Figure S23: Viral vector vaccines versus control (Placebo/Menacxy) on serious adverse events**


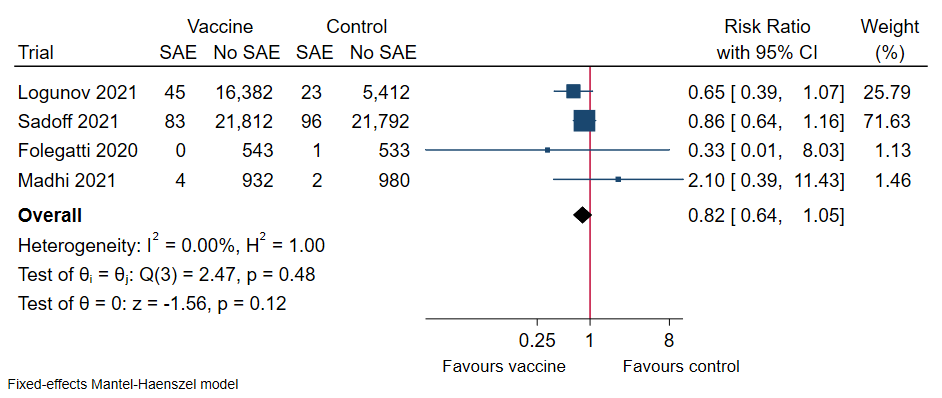


**Figure S24: Trial Sequential Analysis of viral vector vaccines on serious adverse events**


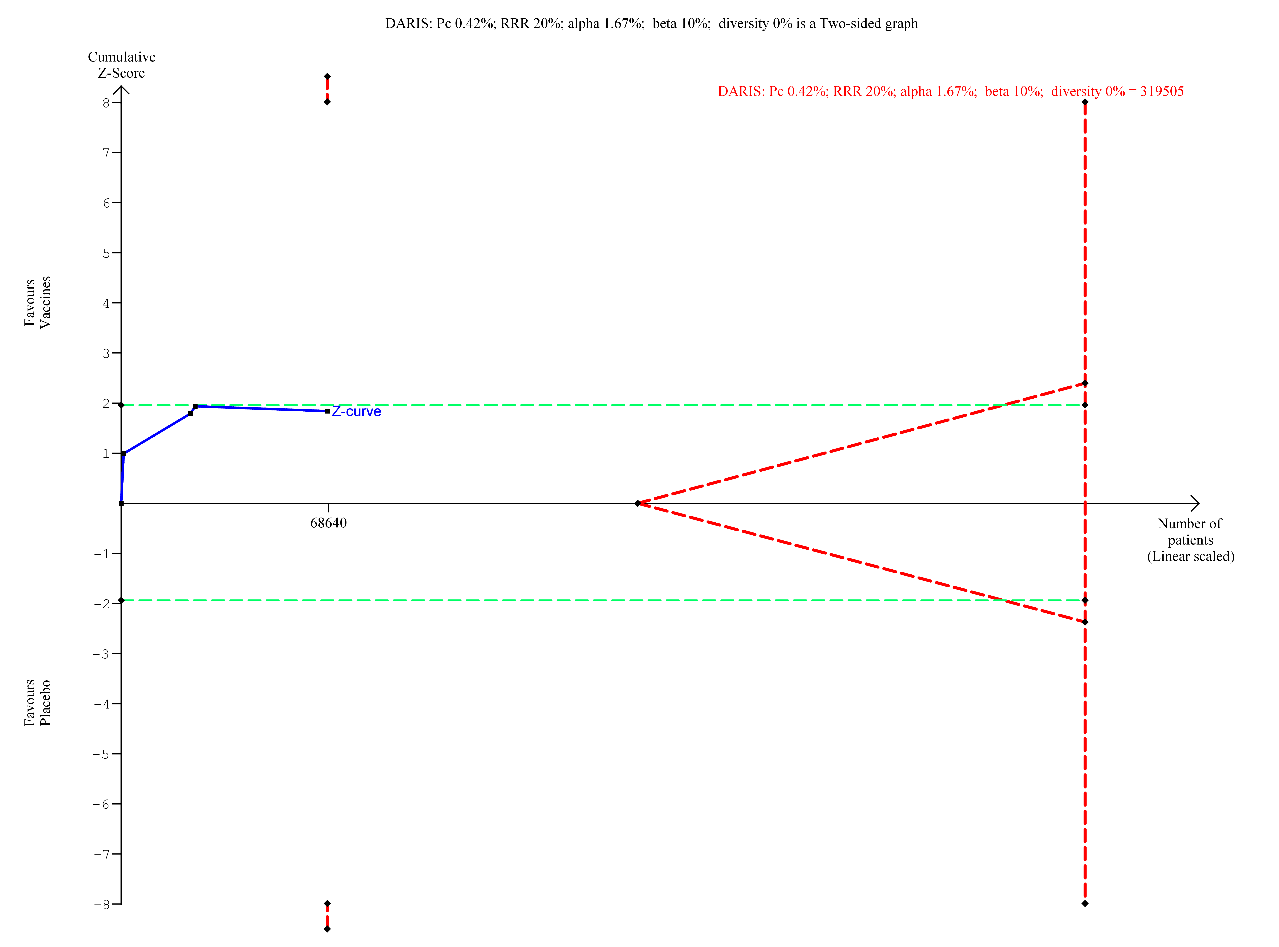


**Figure S25: Inactivated vaccines versus placebo on adverse events considered non-serious**


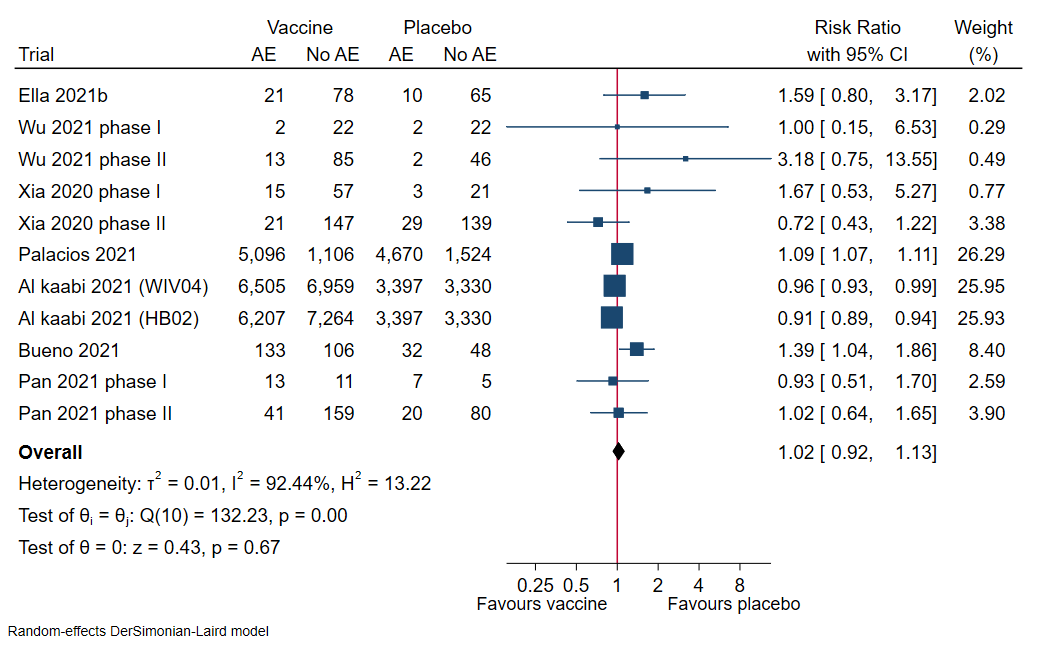


**Figure S26: Trial Sequential Analysis of inactivated vaccines on adverse events considered non-serious**
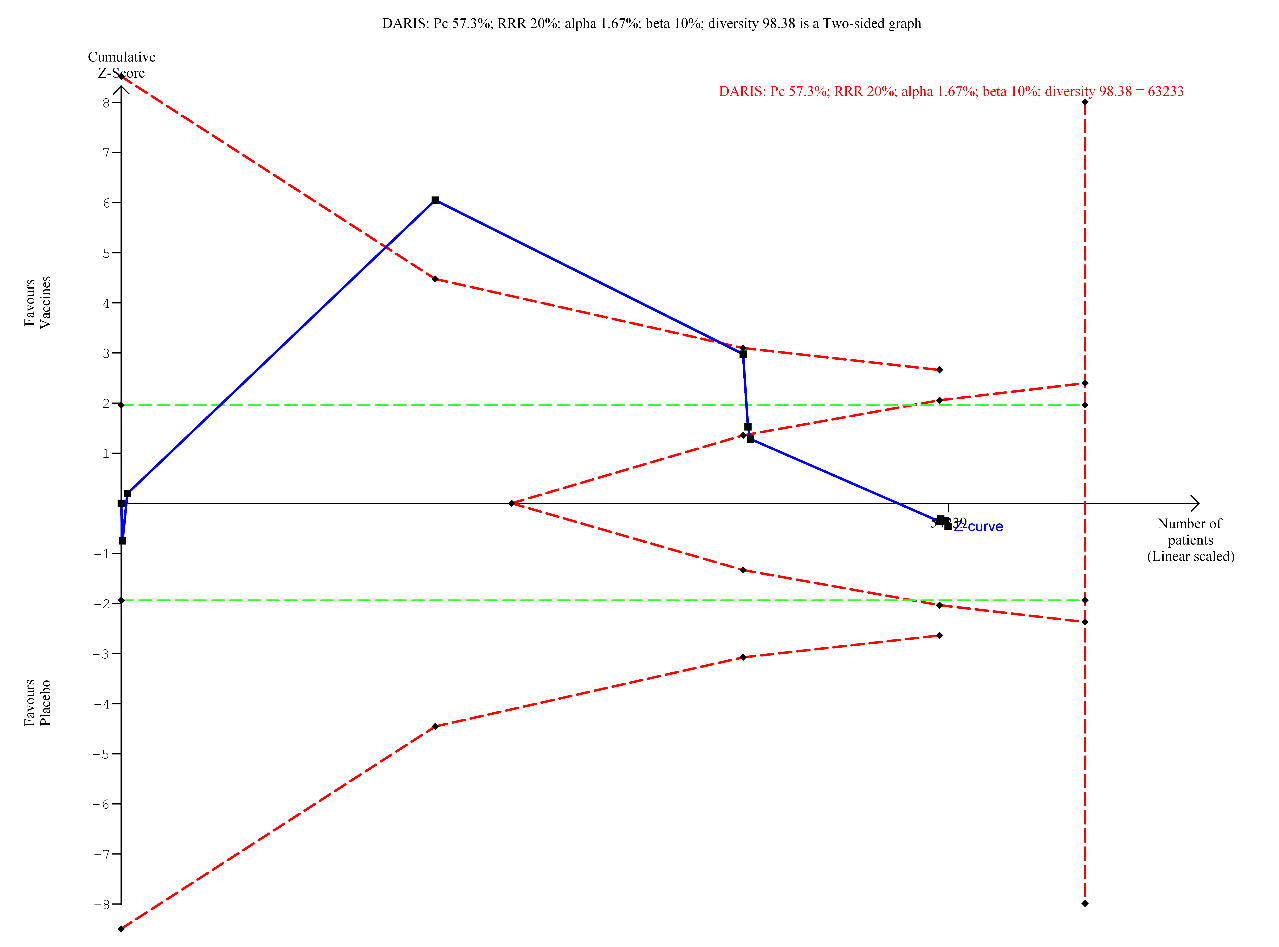


**Figure S27: mRNA vaccines versus placebo on adverse events considered non-serious**


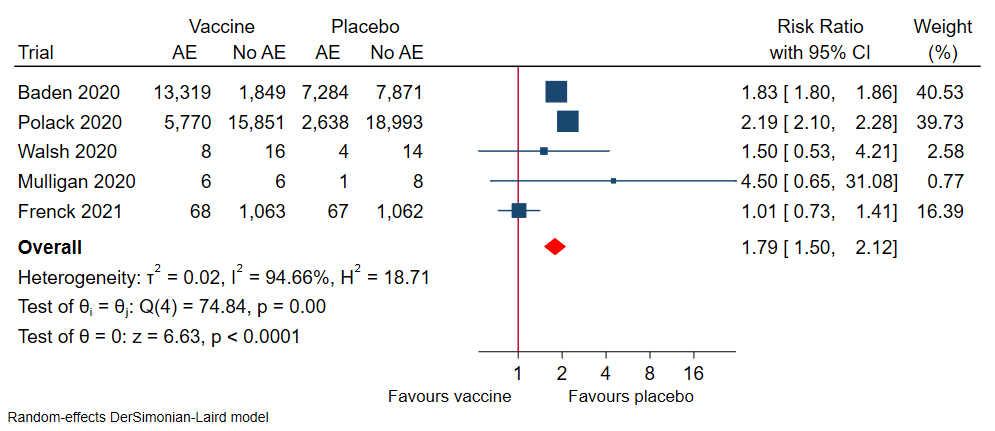


**Figure S28: Trial Sequential Analysis of mRNA vaccines on adverse events considered non-serious**


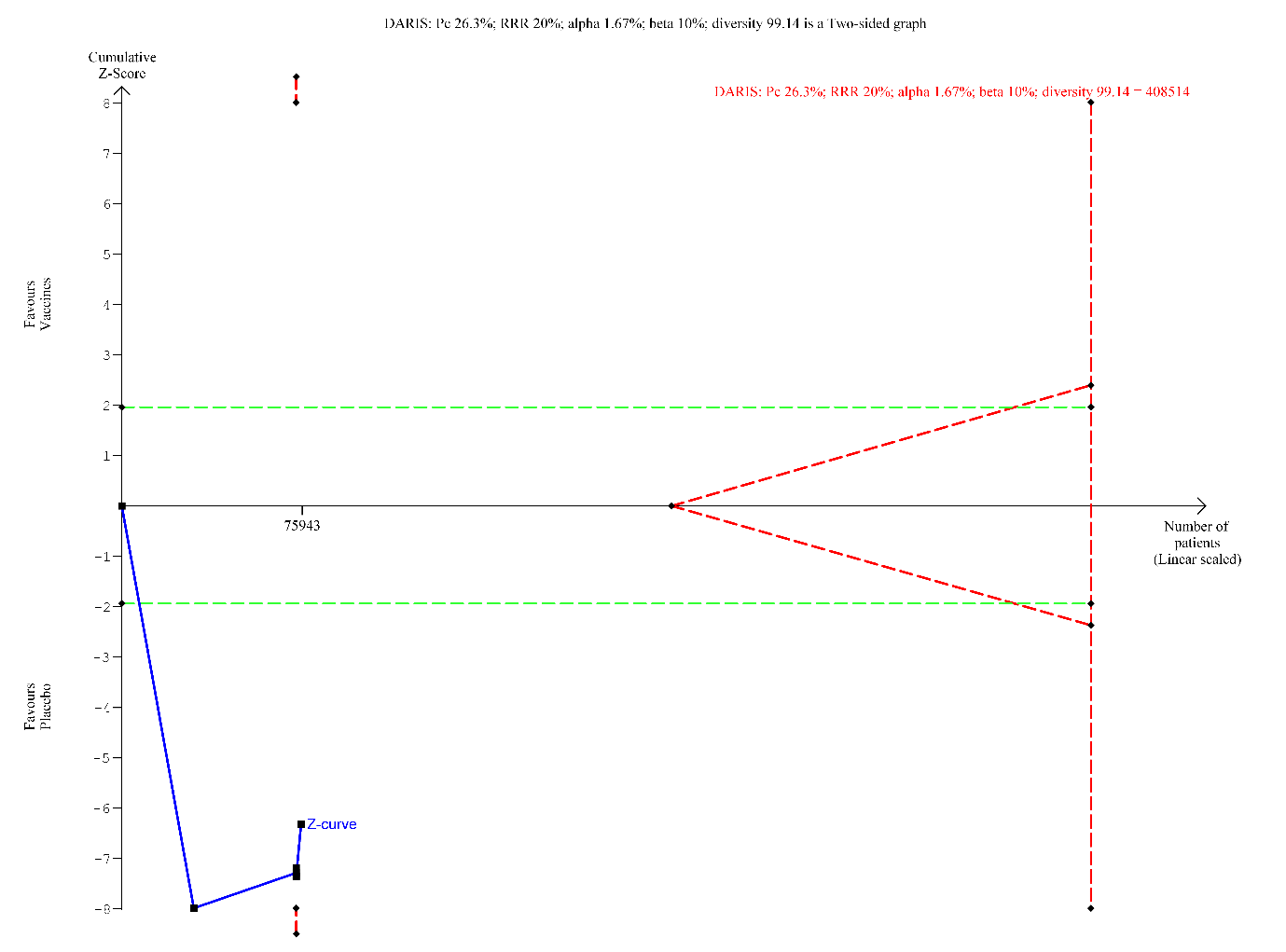


**Figure S29: Protein-subunit versus placebo on adverse events considered non serious**


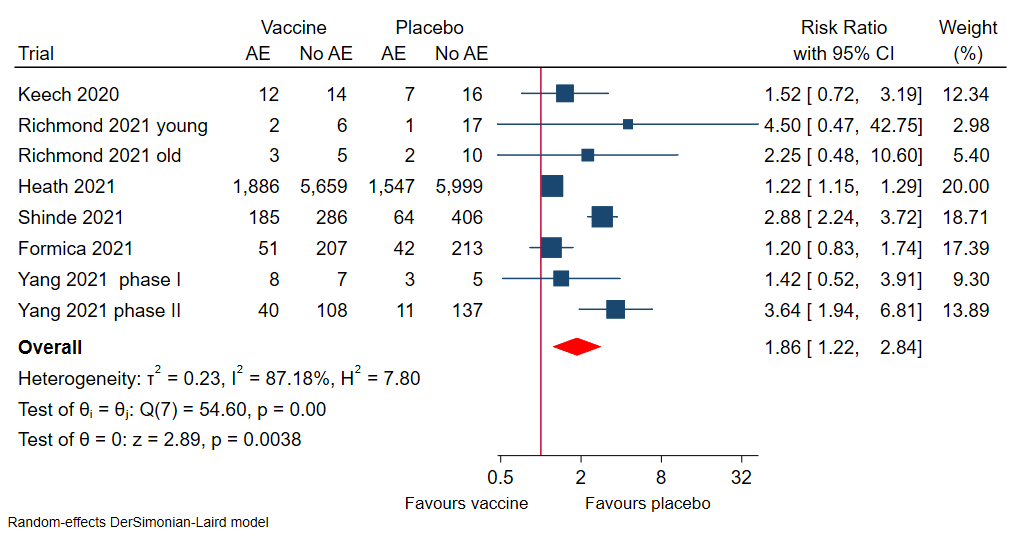


**Figure S30: Trial Sequential Analysis of protein subunit vaccines on adverse events considered non-serious**


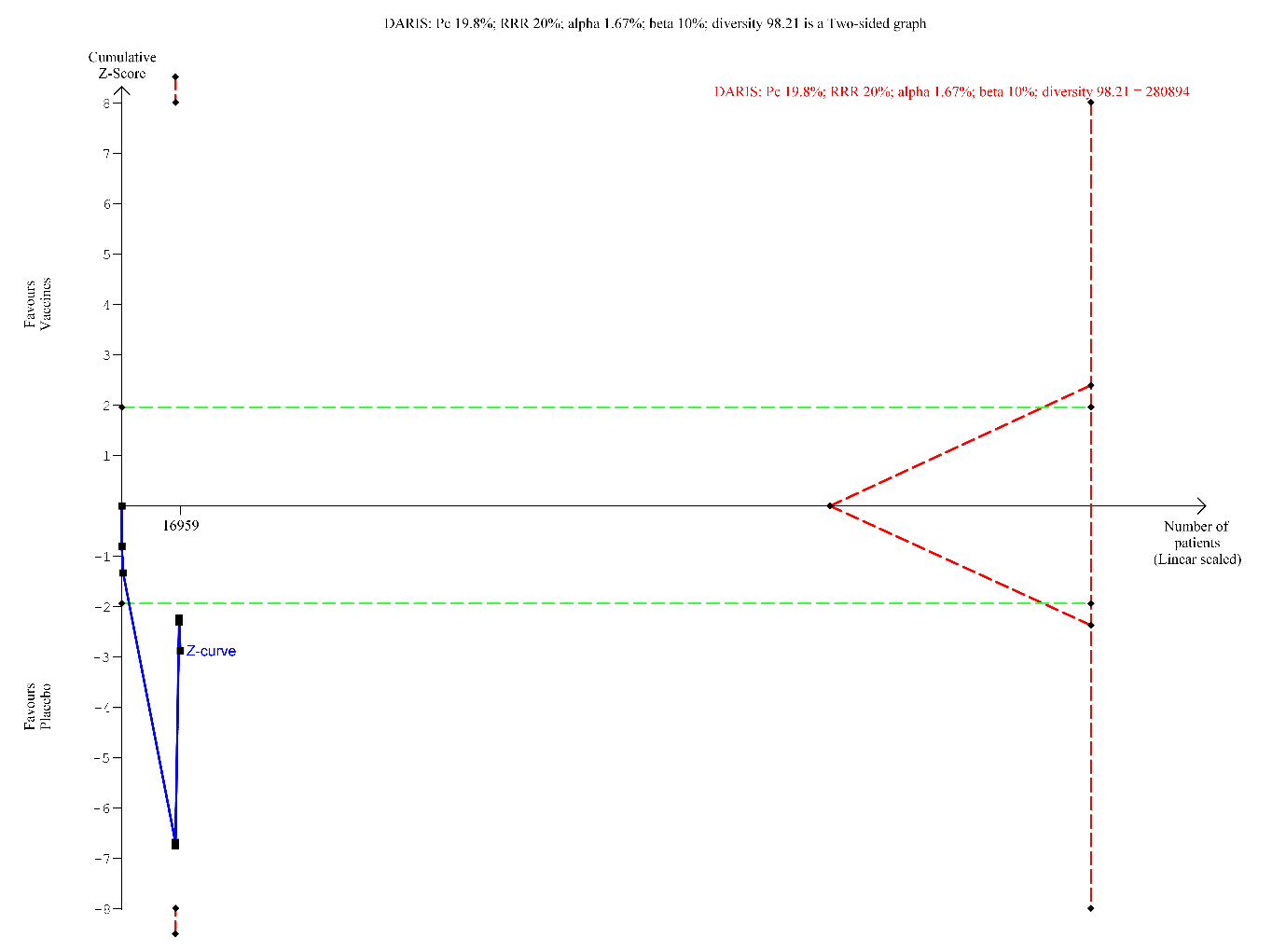


**Figure S31: Viral vector vaccines versus control on adverse events considered non serious**


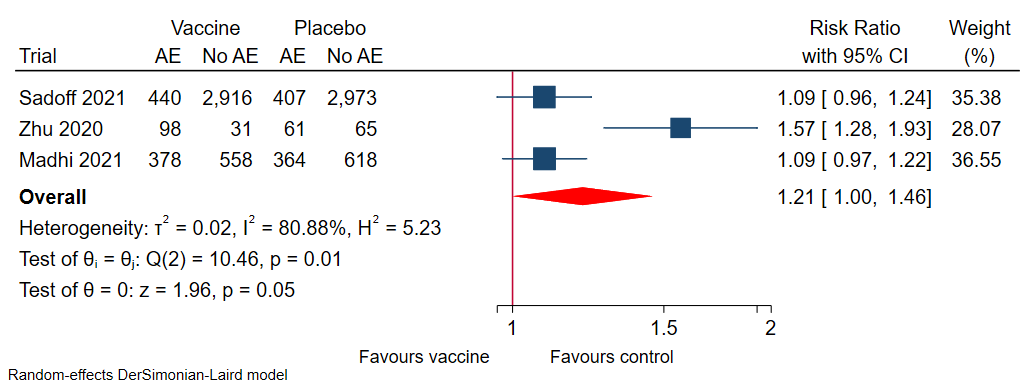


**Figure S32: Trial Sequential Analysis of viral vector vaccines on adverse events considered non-serious**


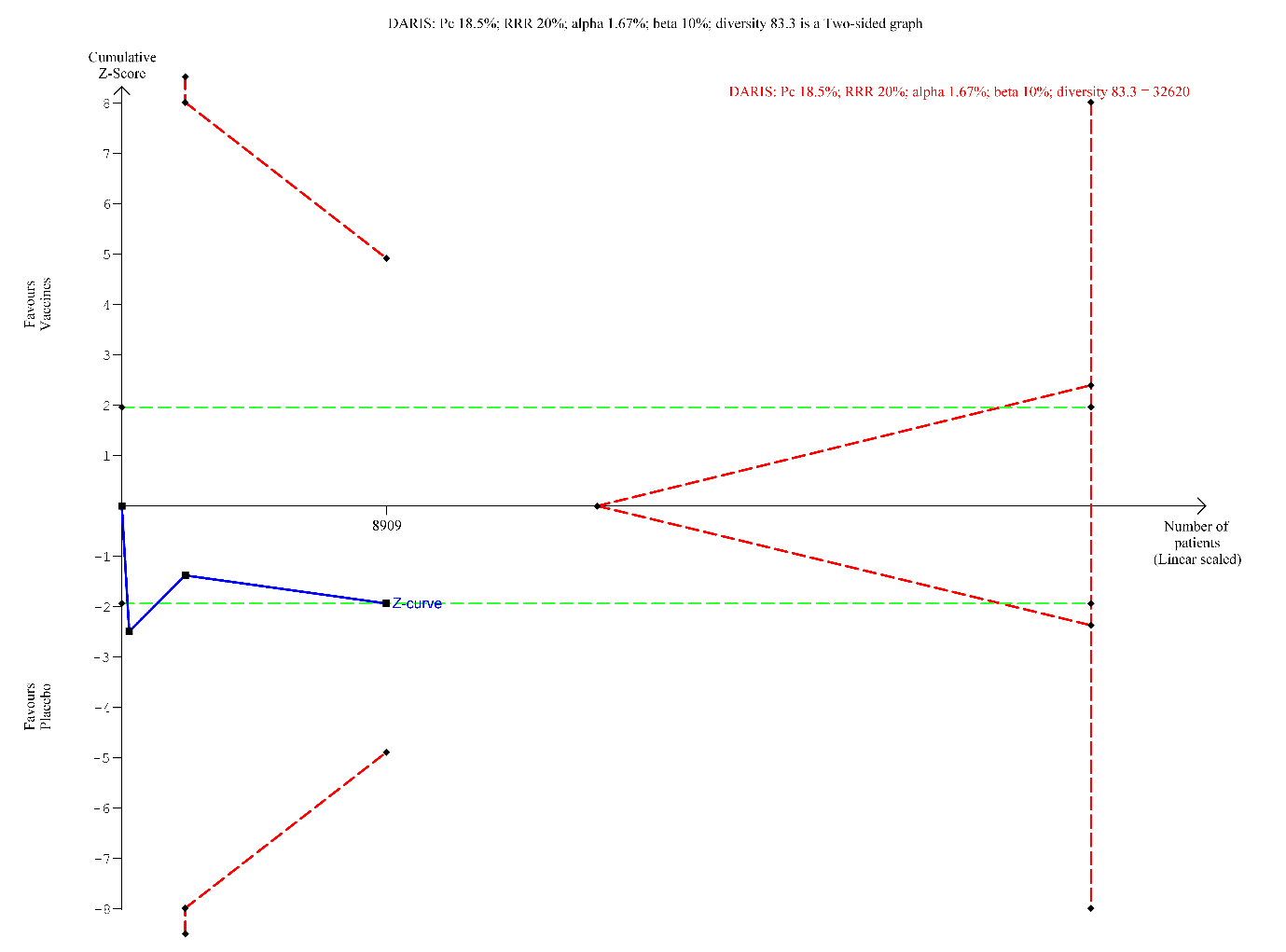


**Figure S25: mRNA vaccine efficacy on prevention of severe COVID-19 plus positive PCR**
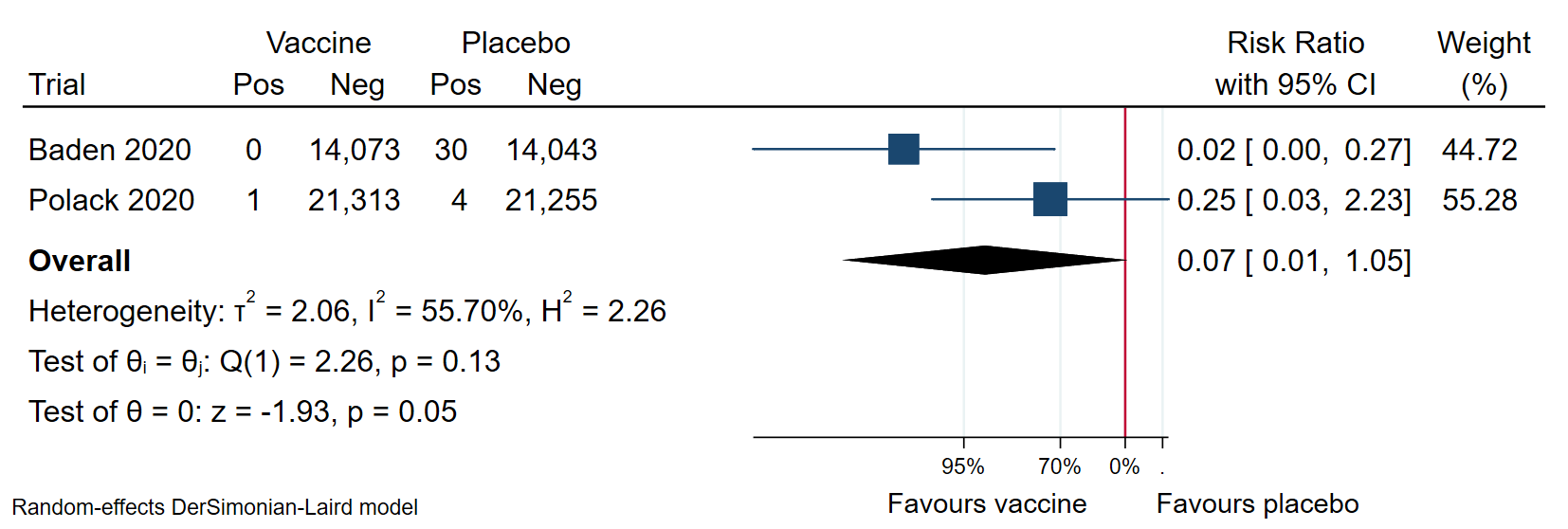


**Figure S26:** **Trial Sequential Analysis on mRNA vaccine efficacy for prevention of severe COVID-19 plus positive PCR**


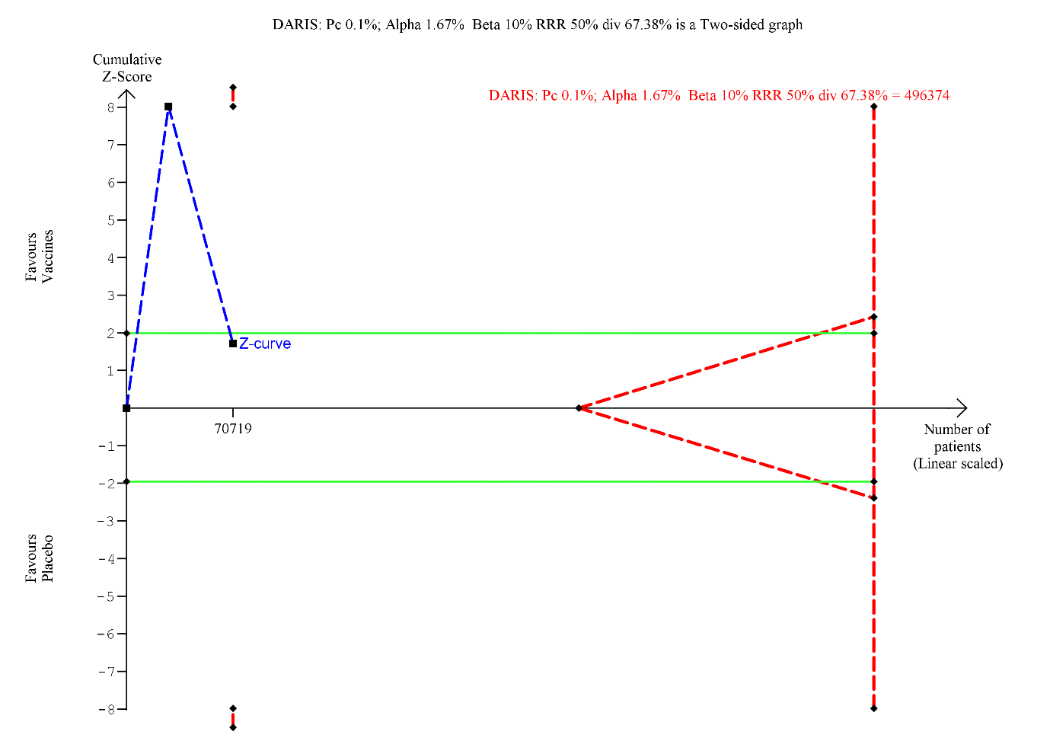


**Figure S27**: **Viral vector vaccine efficacy on prevention of severe COVID-19 plus positive PCR**


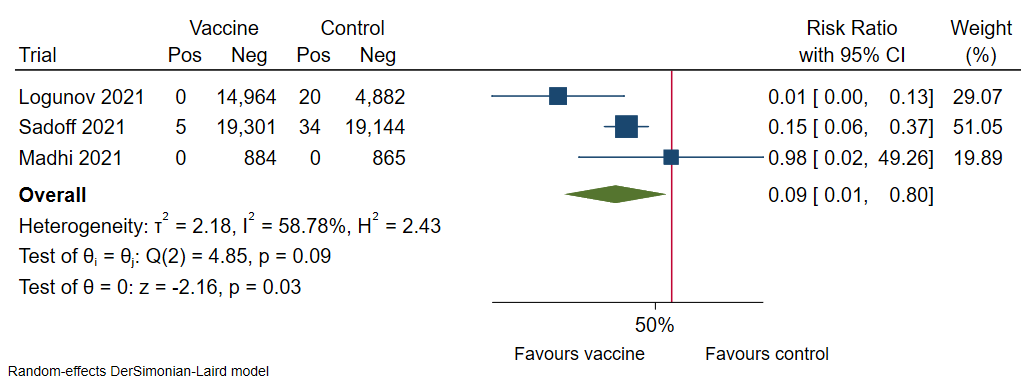


**Figure S28: Trial Sequential Analysis on viral vaccine efficacy on prevention severe test COVID-19 plus positive**


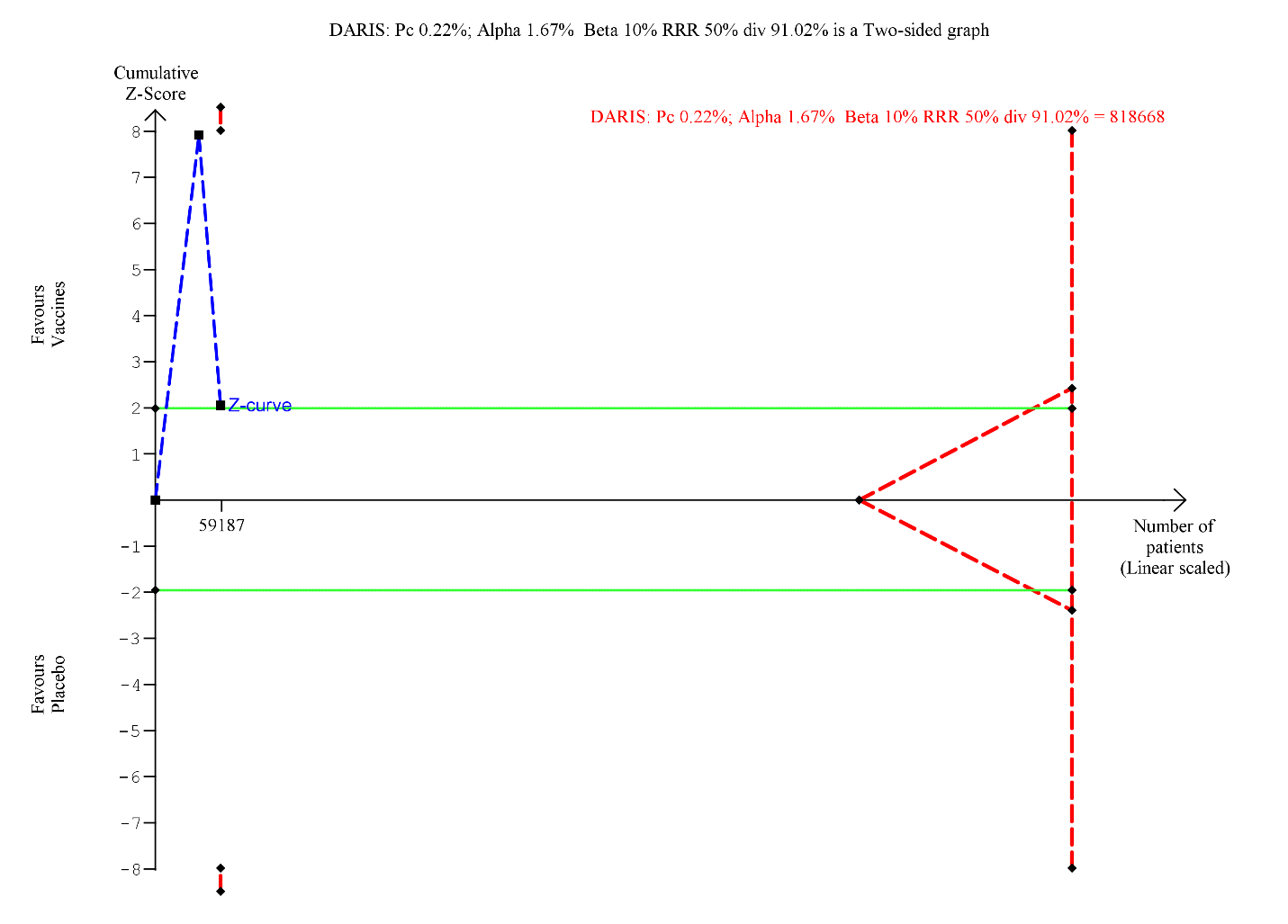


## **Alternative pooling method (random-effects model versus fixed-effect model)**

**Alternative to Figure 1a: Inactivated vaccines versus placebo on all-cause mortality**


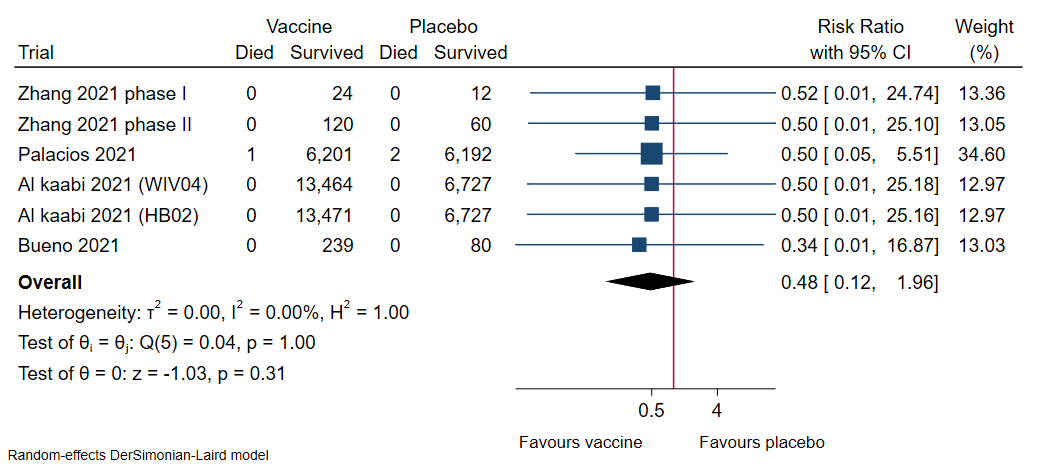


**Alternative to Figure 1b: mRNA vaccines versus placebo on all-cause mortality**


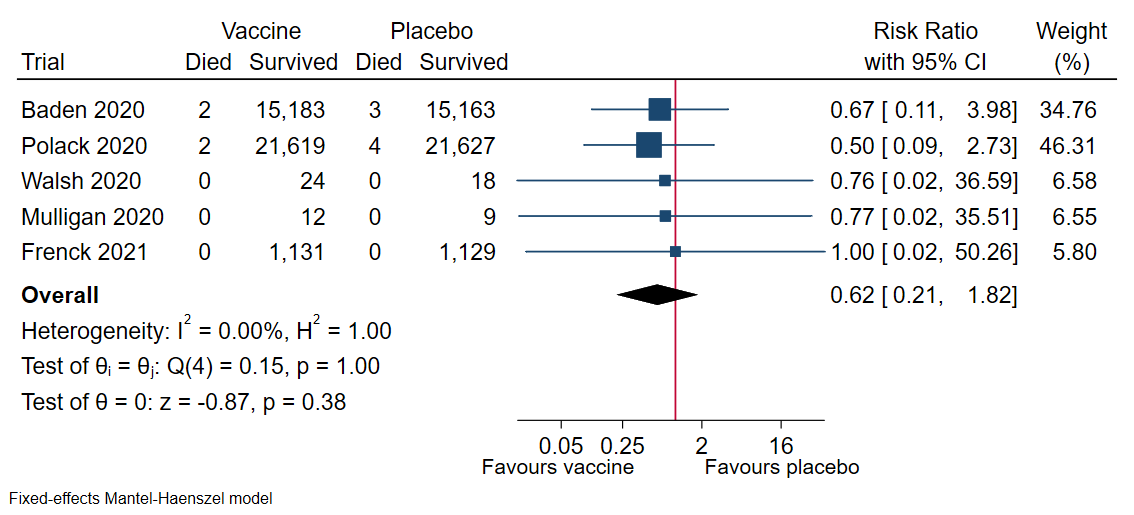


**Alternative to Figure 1c: Protein subunit vaccines versus placebo on all-cause mortality**


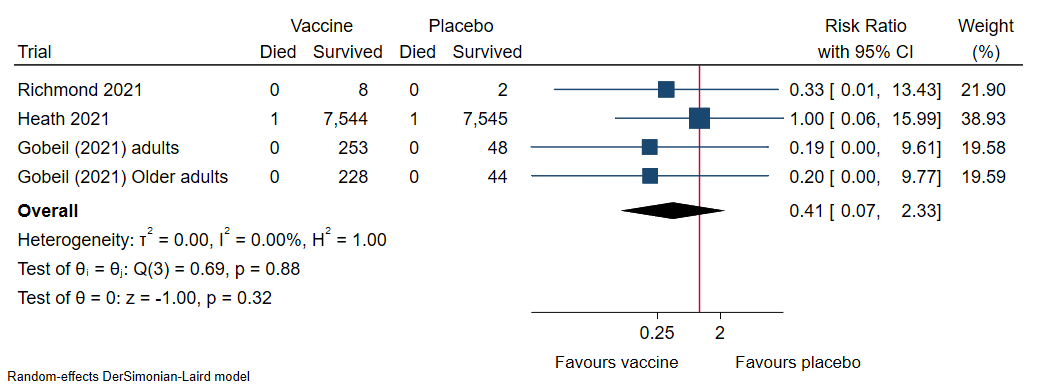


**Alternative to Figure 1d: Viral vector vaccines versus control on all-cause mortality**


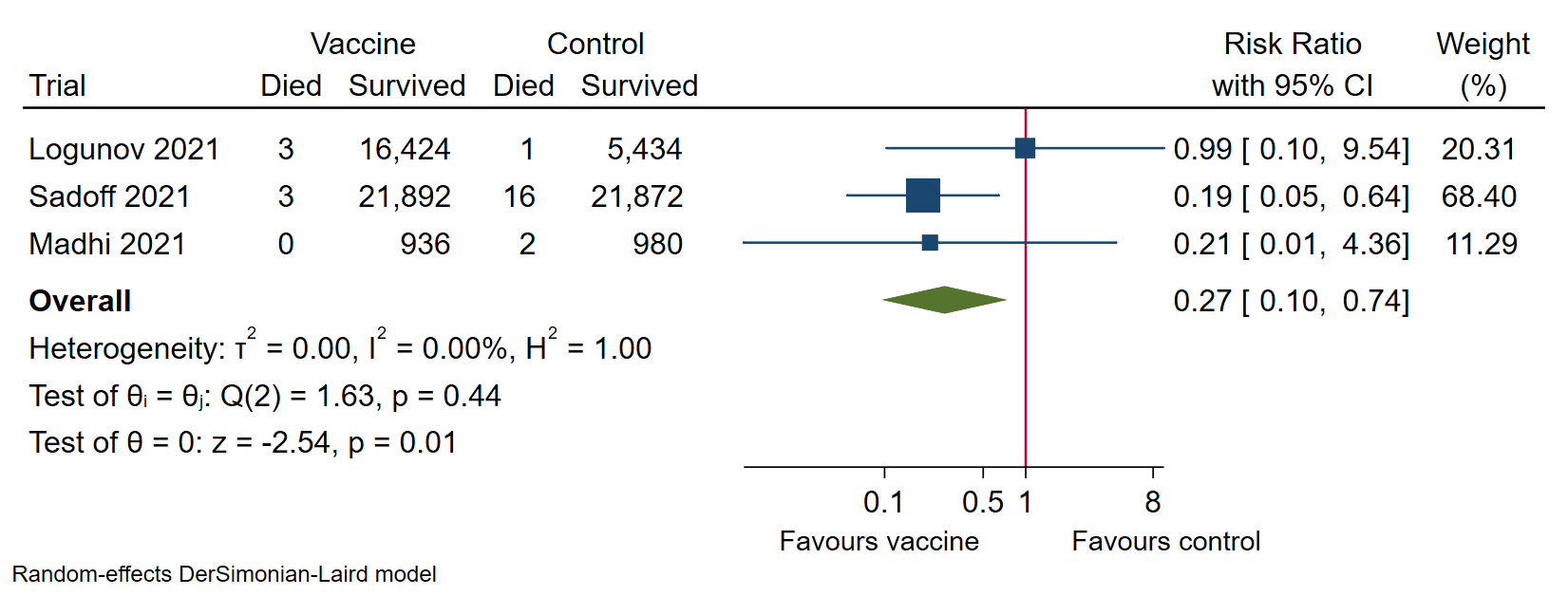


**Alternative to Figure 3a: Inactivated vaccine efficacy on preventing symptomatic COVID-19 plus positive PCR**


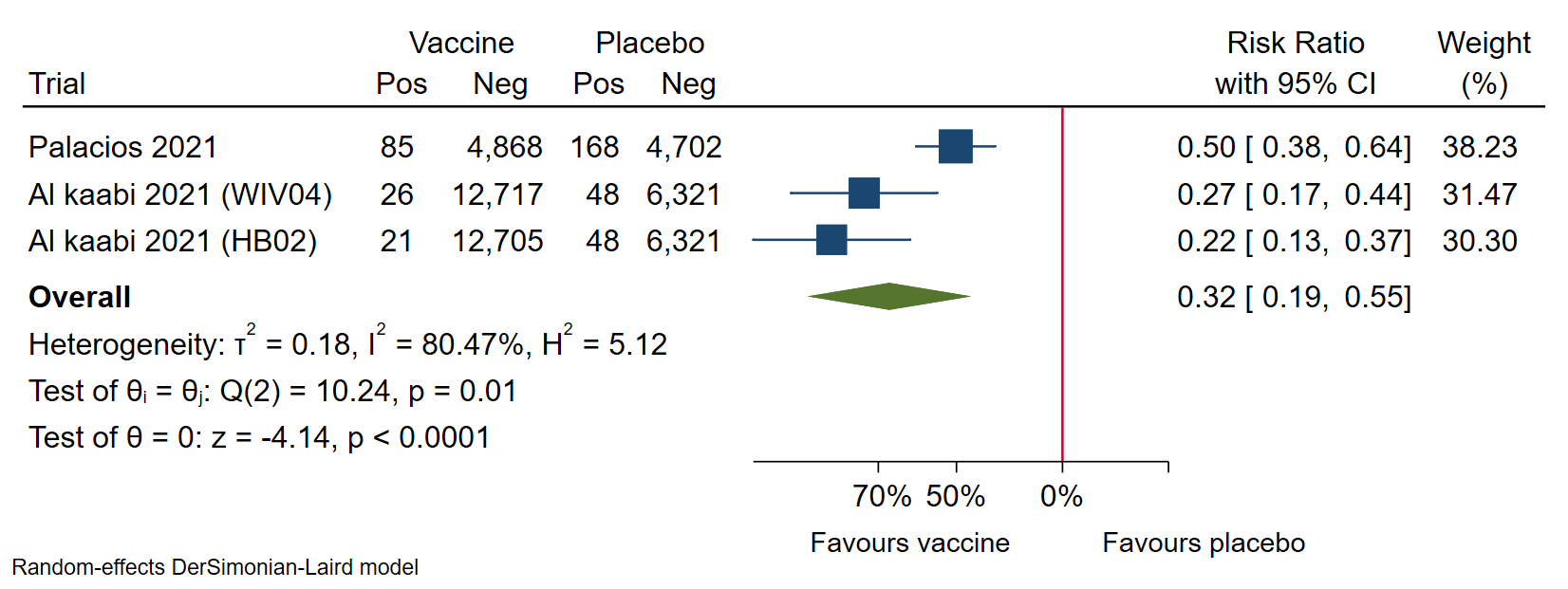


**Alternative to Figure 3b: mRNA vaccine efficacy on preventing symptomatic COVID-19 plus positive PCR**


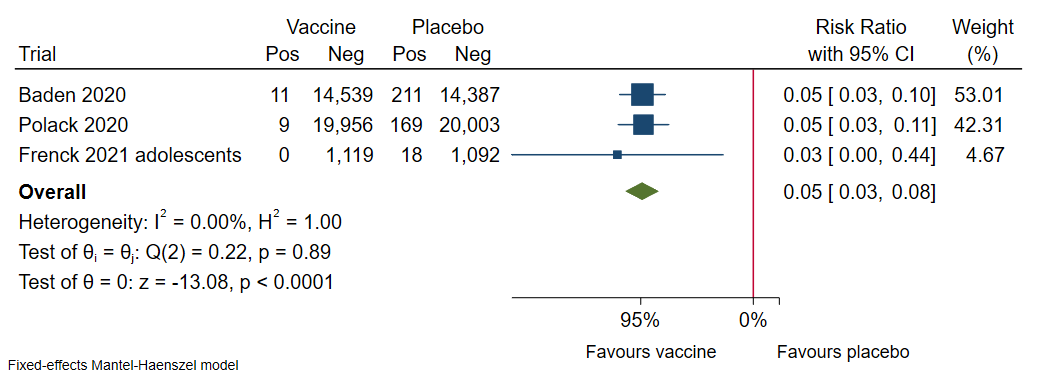


**Alternative to Figure 3c: Protein subunit vaccine efficacy on preventing symptomatic COVID-19 plus positive PCR**


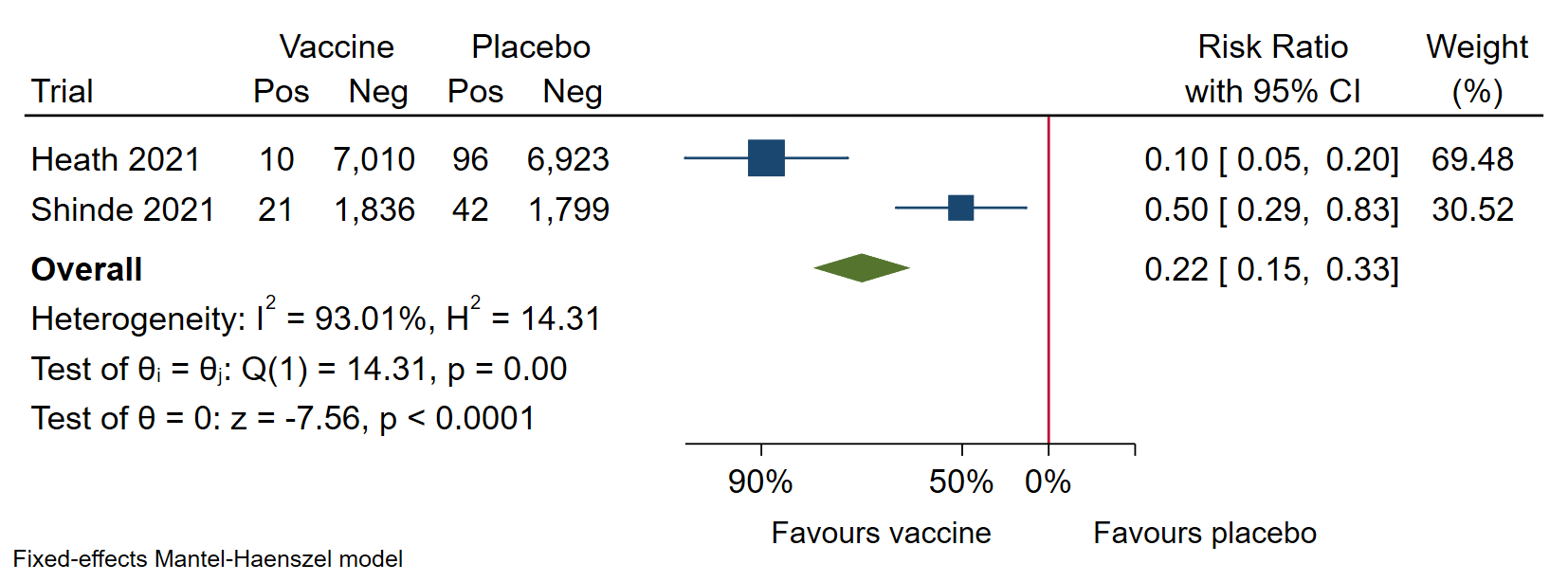


**Alternative to Figure 3d: Viral vector vaccine efficacy on preventing symptomatic COVID-19 plus positive PCR**


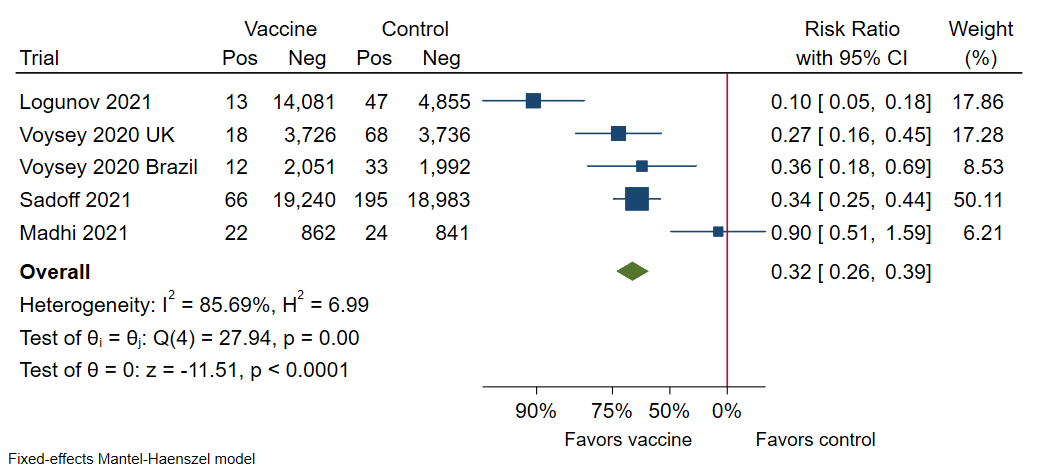


**Alternative to Figure S17: Inactivated vaccines versus placebo on serious adverse events**


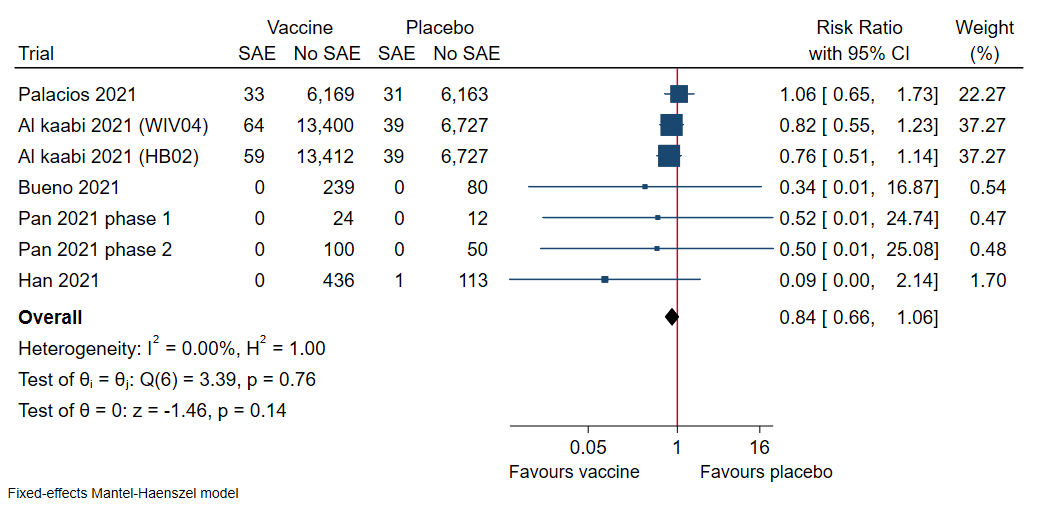


**Alternative to Figure S19: mRNA vaccines versus placebo on serious adverse events**


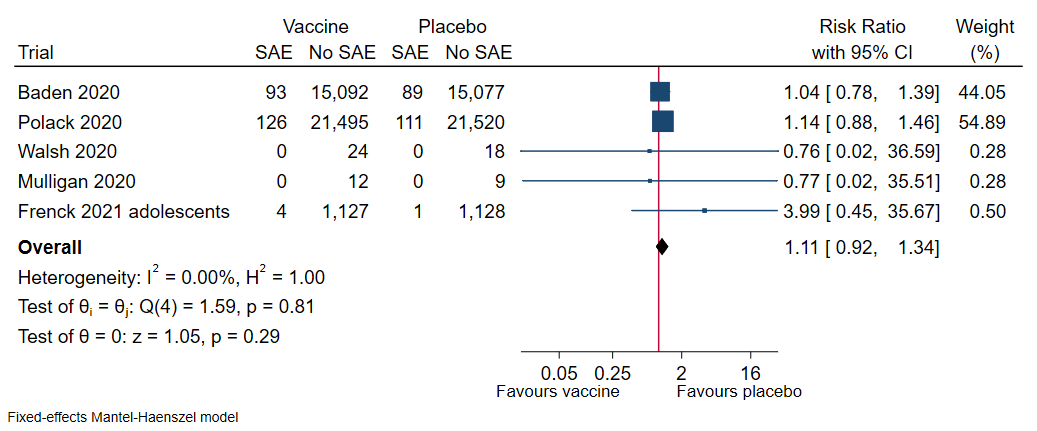


**Alternative to Figure S21: Protein subunit vaccines versus placebo on serious adverse events**


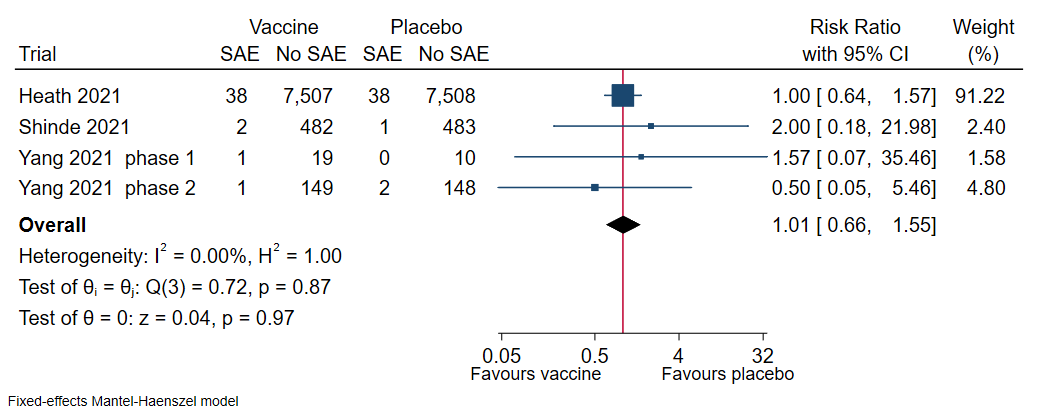


**Alternative to Figure S23: Viral vector vaccines versus control (Placebo/Menacxy) on serious adverse events**


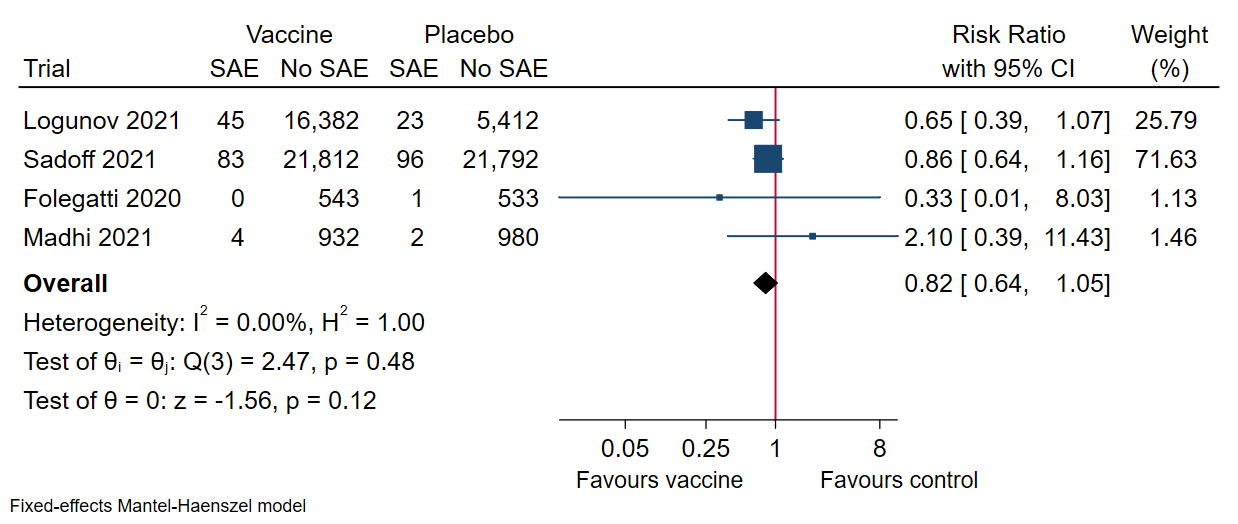


**Alternative to Figure S25: Inactivated vaccines versus placebo on adverse events considered non-serious**


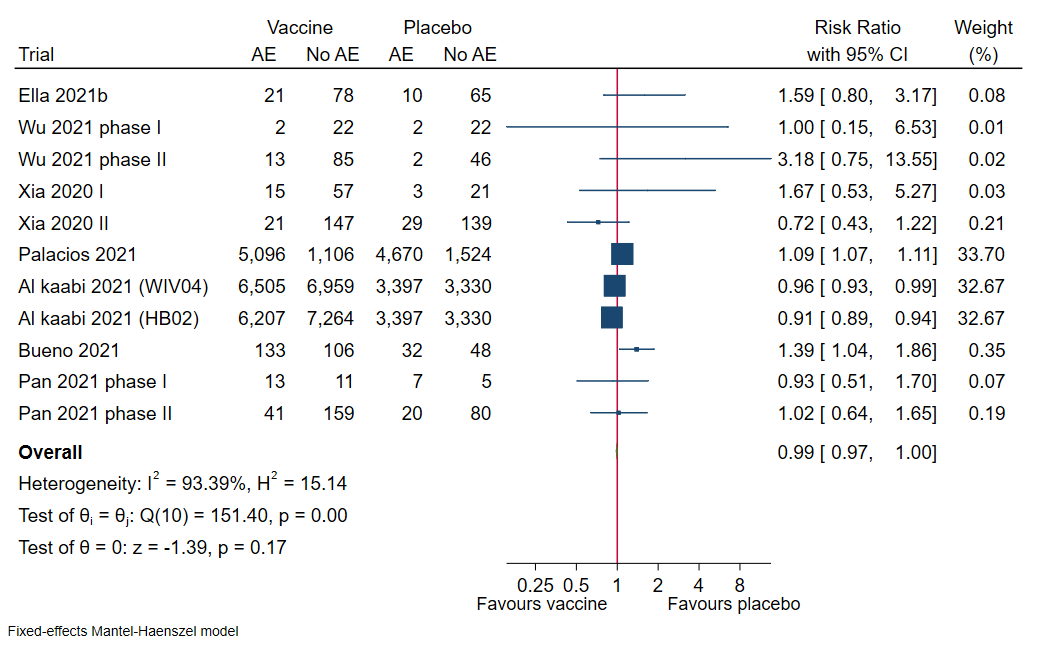


**Alternative to Figure S27: mRNA vaccines versus placebo on adverse events considered non-serious**


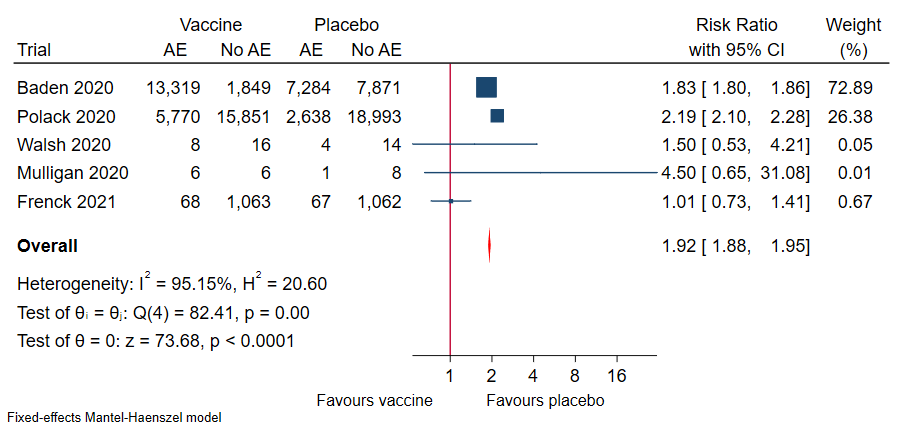


**Alternative to Figure S29: Protein subunit vaccines versus placebo on adverse events considered non-serious**


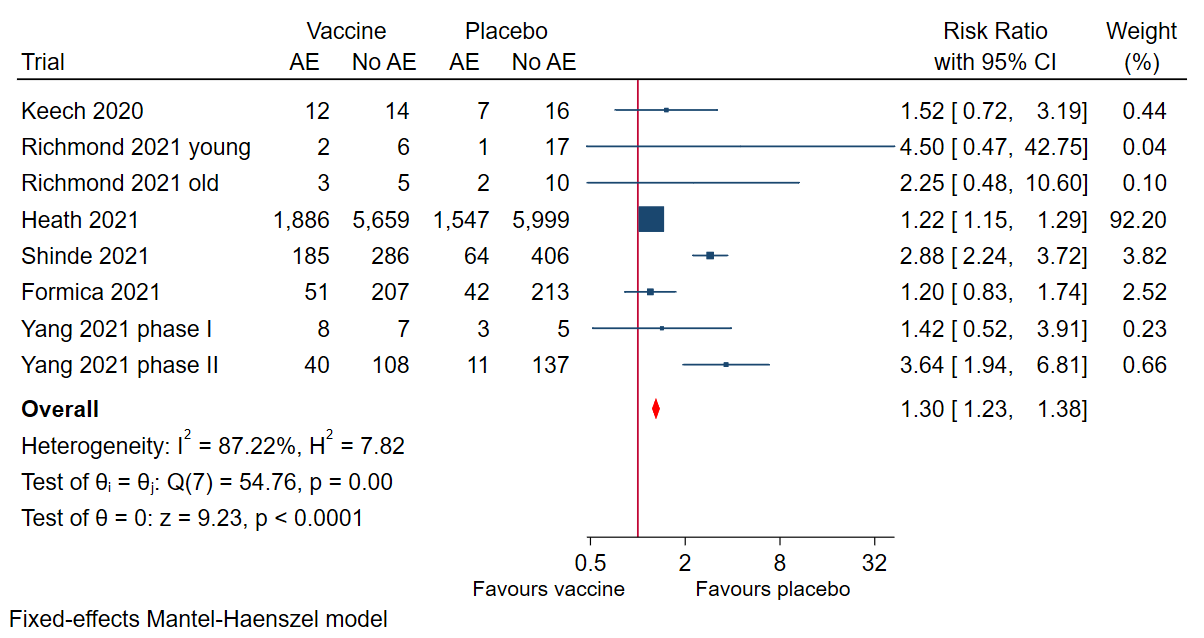


**Alternative to Figure S31: Viral vector vaccines versus control on adverse events considered non serious**


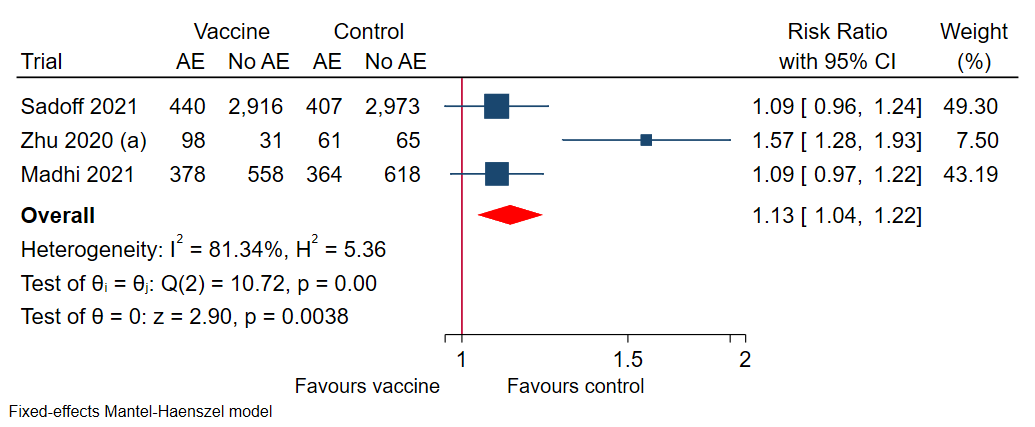


# **References**

1. Korang SK, Juul S, Nielsen EE, Feinberg J, Siddiqui F, Ong G, et al. Vaccines to prevent COVID-19: a protocol for a living systematic review with network meta-analysis including individual patient data (The LIVING VACCINE Project). Syst Rev. 2020 Nov 20;9(1):262.

2. Mills EJ, Thorlund K, Ioannidis JPA. Demystifying trial networks and network meta-analysis. BMJ (Clinical research ed). 2013 May 14;346:f2914.

3. Salanti G, Ades AE, Ioannidis JPA. Graphical methods and numerical summaries for presenting results from multiple-treatment meta-analysis: an overview and tutorial. J Clin Epidemiol. 2011 Feb;64(2):163–71.

4. Shim S, Yoon B-H, Shin I-S, Bae J-M. Network meta-analysis: application and practice using Stata. Epidemiol Health. 2017;39:e2017047.

5. StataCorp. Stata Statistical Software: Release 16 2019 [College Station, TX: StataCorp LLC http://www.stata.com].

6. Veroniki AA, Jackson D, Viechtbauer W, Bender R, Bowden J, Knapp G, et al. Methods to estimate the between-study variance and its uncertainty in meta-analysis. Res Synth Methods. 2016 Mar;7(1):55–79.

7. Rücker G, Schwarzer G. Ranking treatments in frequentist network meta-analysis works without resampling methods. BMC Med Res Methodol. 2015 Jul 31;15:58.

8. Veroniki AA, Straus SE, Fyraridis A, Tricco AC. The rank-heat plot is a novel way to present the results from a network meta-analysis including multiple outcomes. J Clin Epidemiol. 2016;76:193–9.

9. Bakbergenuly I, Kulinskaya E. Beta-binomial model for meta-analysis of odds ratios. Stat Med. 2017 May 20;36(11):1715–34.

10. Günhan BK, Röver C, Friede T. Random-effects meta-analysis of few studies involving rare events. Research Synthesis Methods. 2020;11(1):74–90.

11. Baden LR, El Sahly HM, Essink B, Kotloff K, Frey S, Novak R, et al. Efficacy and Safety of the mRNA-1273 SARS-CoV-2 Vaccine. New England Journal of Medicine. 2020 Dec 30;0(0):null.
